# A Review on the Development of Tunable Graphene Nanoantennas for Terahertz Optoelectronic and Plasmonic Applications

**DOI:** 10.3390/s20051401

**Published:** 2020-03-04

**Authors:** Zaka Ullah, Gunawan Witjaksono, Illani Nawi, Nelson Tansu, Muhammad Irfan Khattak, Muhammad Junaid

**Affiliations:** 1 Department of Electrical and Electronic Engineering, Universiti Teknologi PETRONAS, Bandar Seri Iskandar 32610, Malaysia; zaka_18000817@utp.edu.my; 2 Center for Photonics and Nanoelectronics, Department of Electrical and Computer Engineering, Lehigh University, 7 Asa Drive, Bethlehem, PA 18015, USA; 3 Department of Electrical Communication Engineering, University of Engineering and Technology Peshawar, Kohat campus, Kohat 26030, Pakistan

**Keywords:** graphene antenna, graphene tunable antenna, graphene detectors, optoelectronics, plasmonics, terahertz

## Abstract

Exceptional advancement has been made in the development of graphene optical nanoantennas. They are incorporated with optoelectronic devices for plasmonics application and have been an active research area across the globe. The interest in graphene plasmonic devices is driven by the different applications they have empowered, such as ultrafast nanodevices, photodetection, energy harvesting, biosensing, biomedical imaging and high-speed terahertz communications. In this article, the aim is to provide a detailed review of the essential explanation behind graphene nanoantennas experimental proofs for the developments of graphene-based plasmonics antennas, achieving enhanced light–matter interaction by exploiting graphene material conductivity and optical properties. First, the fundamental graphene nanoantennas and their tunable resonant behavior over THz frequencies are summarized. Furthermore, incorporating graphene–metal hybrid antennas with optoelectronic devices can prompt the acknowledgment of multi-platforms for photonics. More interestingly, various technical methods are critically studied for frequency tuning and active modulation of optical characteristics, through in situ modulations by applying an external electric field. Second, the various methods for radiation beam scanning and beam reconfigurability are discussed through reflectarray and leaky-wave graphene antennas. In particular, numerous graphene antenna photodetectors and graphene rectennas for energy harvesting are studied by giving a critical evaluation of antenna performances, enhanced photodetection, energy conversion efficiency and the significant problems that remain to be addressed. Finally, the potential developments in the synthesis of graphene material and technological methods involved in the fabrication of graphene–metal nanoantennas are discussed.

## 1. Introduction

Innovative electromagnetic optical nanoantennas (NA) functioning at terahertz band, infrared and optical frequencies play a vital role in the emerging field of photonics and plasmonics since these antennas have been considered as the best tools for controlling, manipulation and propagation of light the interaction of light with electrons present in materials [1]. The fundamental capabilities of nanoantennas are vastly utilized in a broad scope of practices, including high-speed communication with high (gigabit/s) data rates in nano-networks, gases detection, inter-chip communication, biosensing of certain chemicals and biological procedures [2], terahertz detection, optical light emission, energy harvesting and optoelectronic devices [3]. These applications have typically activated huge interest and innovative advancement on the ground of nanophotonics in the recent past. At the most progressive principle level, optical antenna devices can be separated into passive and active classes. Passive devices are characteristically linear, for instance, plane optics and light emission, whereas active devices are nonlinear, i.e., photodetection and light energy harvesting [4]. The non-linear attributes of active devices are responsible for their capacity to covert optical light signals into electrical current and to enhance optical signals [5].

Microwave standards are not valid for terahertz region, as the size of the antennas is miniaturized to micrometer scale [6]; thus, the concept of perfect electric conductor (PEC), which is considered in microwave investigations, is not perfect in the range starting from terahertz to optical frequencies, while there are half-wavelength antennas with the size dimension of 1 µm function at 150 THz (i.e., infrared frequency). By manipulating the concept of effective wavelength, conventional antenna configuration can be moved to optical frequencies by employing effective wavelength [7]. However, the antenna devices working the terahertz range (0.1–10 THz), also known as the terahertz gap, have some crucial limitations on their applicability in practical plasmonic and photonic devices [8]. Another issue in the THz range is that the metallic nanoantenna suffers from low antenna efficiency [9], which also bounds its utilization in imaging and spectroscopic applications [10]. The need for new material arises not only in THz antenna devices but optoelectronic operating terahertz gap having enhanced light interaction and reconfiguration of the operating frequency. Various studies have reported that graphene material is the best possible candidate to overcome the limitation of THz. Chen et al. [11] experimentally demonstrated the enhanced emission in the terahertz range by mixing laser radiation with graphene-based photo mixer plasmonic antennas. The work reported in [12] provided enhanced efficiency of the photoconductive antenna using hybrid graphene molybdenum disulfide structure and achieved broad reconfigurability in the THz range. Moreover, graphene in the THz range offers low loss surface plasmon propagation, which endeavors fewer losses than the metallic THz antennas [13]. This property of graphene tunable antennas provides an enhancement in the THz emissions.

The design of optical nanoantenna is analogous to conventional microwave- and millimeter-wave antennas, as it controls the energy conversion of light into localized energy and optical radiation at the subwavelength scale [14]. Various attractive optical systems have two noteworthy dimensional necessities: a microscale optical element and nanoscale radiator [15]. The former is required for the effective coupling of light propagation, while the latter can boost the sensitivity of the device. These antennas couple energy from the high directive far field to the subwavelength localized near-fields. The highly localized and subwavelength behavior of near field in the vicinity of nanoantenna require nanoscale structures matched with the wavelength of light [16]. Typically, the interaction of an antenna with the near-field region is essential, where antenna modes dominate the free space radiation modes. Antenna modes are divided in two; they can be either propagative or evanescent waves. These modes can carry non-radiative and radiative power and even have the ability to accumulate electromagnetic energy [17].

The wavelength of photons is much larger than the wavelength of the electron [18]. Hence, microelectronic devices have concise structures. The transport of electrons is restricted by their mobility or dispersion outcomes, which decreases electron velocity, in contrast with the propagation and transmission of photons. Thus, the dimensions of photonic devices are engineered and limited to the diffraction or half-wavelength [19]. Plasmonic devices can harness light in extraordinary ways; these devices are also overwhelmed by dissipative losses, and the most significant challenge is probably their applicableness in modern photonic devices [20]. In plasmonic devices, the length of the propagating surface plasmon polaritons (SPPs) on the exterior of plasmonic metals is limited due to dissipative losses [21]. These dissipative losses emerge in high electric currents, result in the form of heating due to significant dissipation and the highly electromagnetic confined fields on the surface of metallic materials also bring relaxation losses in the dielectric substrate [22]. It must be kept in mind that the substrate materials constituting nanoantenna have low loss tangent, but some substantial losses still arise due to loss channels excited by high resonant fields [23]. Concentrating on terahertz and higher optical frequencies, the losses are subjugated by heat dissipation on the conductive counterparts even when noble conducting metals are employed. While the noble metals (i.e., gold, silver and alumina) were regarded as best plasmonic conductors for nanoantennas in the past, these metals cannot transmit appropriately because of micro-cracks in the radiative surface. As a result, they act as lossy non-plasmonic conductors in the terahertz and optical regions, which is a crucial obstacle for specific probable applications [24].

Graphene, the wonder material, is an allotrope of carbon having a monoatomic layer of carbon atoms. The carbon atoms in a two-dimensional graphene structure are arranged in a honeycomb lattice due to the bonding of atoms in a hexagon manner. The discovery of graphene has attracted marvelous research curiosity in numerous arenas credited to its distinguishing characteristics [25]. Graphene is not only famous and valuable in material science and quantum matter physics but also in optoelectronics and photonic device societies [26]. In contrast with other more conventional materials, for example, silicon and III–V semiconductors, graphene exhibits exceptional and outstanding properties, having high electron mobility, enormous thermal conductivity, robust mechanical flexibility and high third-order optical nonlinearities [27] Nonetheless, the utilization of graphene is less studied in the field of nanoantennas and passive optical guided devices. The structural dimensions of these antennas need to be in the order of the wavelength relative to the electrical length, while the graphene samples available have a much smaller size [28]. In contrast to the above statement, advanced graphene synthesis methods such as chemical vapor deposition (CVD) and plasma chemical vapor deposition (PCVD) make it possible to grow graphene samples to quite a few centimeters, thus increasing the research interest of the passive nanoantenna devices at such high frequencies [29]. The featured list of investigations is presented in various plasmonics domains. For example, graphene-integrated plasmonics, metamaterials, high-speed transistors and crystal clear solar cells are among the most recognized applications [30]. Future directions based on integration of 2D materials and III-V (or III-nitride) technologies will be highly exciting. 

Graphene displays high wave localization and can underpin the propagation of SPPs, with acceptable loss. It also portrays a remarkable tunability through doping of certain chemicals and by electric/magnetic biasing [31]. Significantly, at terahertz and infrared frequencies, plasmonic responses appear in graphene. Thereby, graphene has immediately situated itself as an exceptionally encouraging platform for terahertz transmitters/receivers and antenna integrated optoelectronics [32]. While SPPs can be upheld by composite structures at optical and terahertz frequencies, graphene is becoming considered a flawless material to deliver plasmonic responses, hence opening exciting and unforeseen scenarios intended for lightwave manipulation and radiation at optical frequency range [33]. The unique features of graphene are already exploited in nanoantennas such as reflectarray antennas [34], frequency reconfigurable antennas [35] and leaky-wave antennas with the best performance and high radiation efficiency [36]. Graphene antennas resonate at much lower frequencies relative to their metallic counterparts and can exploit the tunable conductivity behavior of graphene complex [37].

All essential components of optical transceivers can utilize graphene plasmonics: optical switches, optical waveguides and modulators phase shifters. Furthermore, biasing of graphene through magneto-static fields has been demonstrated to result in giant Faraday rotation and non-reciprocal propagation [38,39]. More interestingly, exceptional graphene characteristics have promoted the advancement in magnet-free non-reciprocal guided systems and nanoantennas grounded on spatiotemporal modulation of conductivity, permitting to break the significant standard of time inversion symmetry without using ferromagnetic materials [40]. Satisfactory integration of optical nanoantenna with important optical components functioning in a single photonic device will fundamentally push the limits of current terahertz innovations with extraordinary achievements and behaviors in various applications such as silicon-adaptable terahertz communication, optical energy conversion and infrared sensing [41].

It is worth noting that, to date, graphene-based photonics devices (i.e., antennas and photodetectors) have only been proposed and scrutinized hypothetically, and the electromagnetic response evaluated through simulators by using numerical solvers [42,43,44,45]. However, the ongoing experiments by the research community has completely confirmed the propagation of localized and tunable SPPs in graphene layers, which is a physical phenomenon and all nanoantenna devices portrayed in the following sections rely on surface plasmonic effect. Previous investigations produced graphene of a deficient quality, which was not suitable for an electromagnetic application other than to work as a variable resistor, restricting the applied viability of research work [46,47,48]. However, in the last few years, tremendous improvements have been observed, because of the comprehensive hard work by researchers to promote the advancement of fabrication procedures. For example, mobility has been enhanced to many orders of magnitude by sandwiching graphene nanostructures between the layers of boron nitride [49,50,51]. Recently, despite the difficult fabrication and characterization methods involved, the response of some nanoantenna terahertz detectors and frequency reconfigurable arrays have been experimentally proved, obviously performing better than the previous ones but also in good agreement with numerical simulations [52,53]. These groundbreaking experimental works have marginally uncovered pieces of the enormous capability of plasmonics, utilizing 2D graphene and other materials [54,55,56]. Thus, the use of graphene and other 2D materials in the nanoantenna field appears more brilliant now than during its initial advances. However, this field, being at its early stage, will experience exploration to meet the current demands of nanoantennas in the field of plasmonics.

In this article, state-of-the-art graphene plasmonic nanoantennas and hybrid metal–graphene nanoantennas are discussed in detail. The latest techniques in the development of graphene nanoantennas for photonics applications are identified. In Section 2, a structural model of graphene and the factors affecting graphene conductivity are studied at terahertz frequencies. In Section 3, some of the important graphene resonant antennas are analyzed based on their performance and behaviors when graphene is used as a radiating element. In addition, the comparative study between metallic and graphene antennas is discussed. The tunability of graphene nanoantennas is systematically studied in Section 4. Reflectarray and leak-wave graphene antennas having beam steering and beam scanning properties are discussed in Section 5. In Section 6 and Section 7, graphene antenna photodetector and graphene rectennas for energy harvesting application are studied. The fabrication of graphene–metal antenna and development of graphene synthesis are detailed in Section 8.

## 2. Structural Foundation and Modeling of Graphene

**Figure 1:**
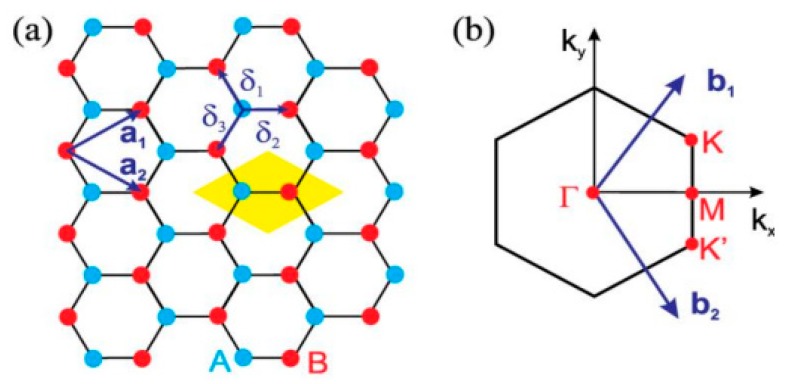
(**a**) Graphene hexagonal lattice, where unit vectors a_1_ and a_2_ represent sublattice and two atoms per unit cell, correspondingly. The bond length between the two nearest neighbor atoms is δ. Blue arrows represent lattice vectors. (**b**) b_1_ and b_2_ represent reciprocal lattice vectors at the Brillouin zone. © 2018 Springer Nature. Reprinted with Permission From [57].

Graphene is a single atomic thick sheet of carbon atoms arranged in a hexagon manner [57], as illustrated in Figure 1. The electromagnetic response of graphene is much reliant on its electronic structure. The Hamiltonian of graphene can be described by tight-binding model theory, as well as creation and annihilation vectors [58], and is written as

(1) $$H=-t\sum_{i}a_{r_{i}}^{+}b_{r_{i}+e_{1}}-t\sum_{i}a_{r_{i}}^{+}b_{r_{i}+e_{2}}-t\sum_{i}a_{r_{i}}^{+}b_{r_{i}+e_{3}}+h.c.,$$

The vectors $e_{1},\;e_{2},$ and $e_{3}$ are directed to their nearest-neighbor sites. The length between the two most adjacent atoms, “*a*”, is also called lattice constant. The hopping energy between the most adjacent atoms is *t* ≈ 2.8 eV. The Hamiltonian can be written as a series of summations in space as follows:

(2) $$H=-t\sum_{k}a_{k}^{+}b_{k}\left(e^{-ik.e_{1}}+e^{-ik.e_{2}}e^{-ik.e_{3}}\right)+h.c$$

where $a(\vec{k})=1+e^{-i\vec{k}.\vec{a_{1}}}+e^{-i\vec{k}.\vec{a_{2}}}$. The eigenvalue problem can be finally formulated as [57]:

(3) $$\left(\begin{array}{cc} E\left(\vec{k}\right) & \alpha (\gamma_{0}E\left(\vec{k}\right)-\gamma_{1})\\ \alpha^{*}(\gamma_{0}E\left(\vec{k}\right)-\gamma_{1}) & E\left(\vec{k}\right) \end{array}\right)\left(\begin{array}{c} a_{k}\\ b_{k} \end{array}\right)=\left(\begin{array}{c} 0\\ 0 \end{array}\right)$$

For γ_0_ = 0 and γ_1_ = 0, the solution is simply $E\left(\vec{k}\right)$ = 0. The dispersion relation $E\left(\vec{k}\right)$ is derived from Equation (3) by putting determinant to zero.

(4) $$E(\vec{k})=\pm \gamma_{1}\left|a(\vec{k})\right|$$

The magnitude of α can be calculated through Equation (5). The result obtained from Equation (5) is often expressed in a different form by using (x, y) components for $\vec{k}$, as shown in Equation (6).

(5) $$E(\vec{k})=\pm \gamma_{1}\sqrt{3+2\cos(\vec{k}.{\vec{a}}_{1})+2\cos(\vec{k}.{\vec{a}}_{2})+2\cos(\vec{k}.({\vec{a}}_{2}-{\vec{a}}_{1}))}$$

(6) $$E\left(k_{x}.k_{y}\right)=\pm \gamma_{1}\sqrt{1+4\cos\left(\frac{\sqrt{3a}k_{y}}{2}\right)\cos\left(\frac{ak_{x}}{2}\right)+4\cos^{2}\left(\frac{ak_{x}}{2}\right)}$$

At first Brillouin zone, six Dirac points occur due to contact and the short distance between conduction and valance bands. By employing a Taylor expansion series at these Dirac points, the graphene dispersion relation is estimated as linear:

(7) E(k)=±ℏvF|k|, ℏvF=3at/2,

where V_F_ is the Fermi velocity roughly equal to ≈10^6^ m/s.

The mobility of electrons in graphene at the conduction band is also referred to as intraband transition. The intraband transition according to Boltzmann equation can be calculated as

(8) $$\frac{df}{dt}+\frac{dp}{dt}\cdot \frac{dE}{dp}\frac{df}{dE}=\frac{f_{o}-f}{\tau }$$

The kinetic energy and momentum of the electrons are represented by *E* and *p*, respectively, and the electron probability density function is denoted by *f* in k space. Here, *τ* is used for relaxation time and time approximation. Equation (8) can be rewritten as Equation (9) by applying a Fourier transform.

(9) $$\left(-i\omega +\frac{1}{\tau }\right)=-F.v\frac{df}{dE}+\frac{f_{o}}{\tau }$$

In Equation (9), group velocity is represented by *v* and the electric field vector is denoted by *E*. *E = hv* and *v = dw/dk*. Finally, integrating Equation (9) by *v* and then again at *k*-space, the following equation is obtained:

(10) $$\frac{g}{{\left(2\pi \right)}^{2}}\left(-i\omega +\frac{1}{\tau }\right)\int efvdk_{x}=\frac{-eg}{{\left(2\pi \right)}^{2}}\int F.\left(vv\right)\frac{\delta f}{\delta E}dk_{x}dk_{y}$$

In a unit cell, two carbon atoms and spin degeneracy “*g*” are equivalent to four for the graphene layer considered in Equation (10).

Manipulating $J=g/{\left(2\pi \right)}^{2}\int^{\;}efvdk_{x}dk_{y}$ in Equation (10), the following relation of current for conduction-band is attained:

(11) $$J=\frac{1}{-i\omega +\frac{1}{\tau }}\frac{e}{\pi^{2}}\int F.(\nu \nu )\frac{-\delta f}{\delta E}dk_{x}dk_{y}$$

In the following, Hall impedance, which occurs due to a magnetic field is ignored and electrical impedance is considered, that is,

(12) $$\begin{array}{l} \sigma_{xx}^{\mathrm{int}ra}=\frac{1}{-i\omega +\tau^{-1}}\frac{e^{2}}{\pi \hbar^{2}}\int \frac{-\delta f}{\delta E}EdE=\frac{-i}{\omega +i\tau^{-1}}\frac{e^{2}}{\pi \hbar^{2}}\int_{0}^{\infty }E\left[\frac{-\delta f(E)}{\delta E}-\frac{\delta f(-E)}{\delta E}\right]dE \end{array}$$

Equation (12) agrees with Kubo formalism for the intraband transition part. The unique electronic band structure of graphene directly affects the conductivity of graphene, as well as the other parameters, for instance, chemical potential, chemical doping, relaxation time, electrons mobility and the surrounding temperature operating frequency. In addition, these characteristics parameters are correlated with each other [59,60,61]. At terahertz (THz) frequencies, the conductivity of graphene can be modeled by using the intraband part of Kubo equation and employing classical and local models without magnetic bias, as can be applied for higher frequencies using the interband part:

(13) $$\sigma^{\mathrm{int}ra}=-j\frac{e^{2}k_{B}T}{\pi \hbar^{2}(\omega -/\tau^{-1})}\ln\left\{2\left[1+\cosh\left(\frac{\mu_{c}}{k_{B}T}\right)\right]\right\}$$

where *e* represents the charge on the electron, angular frequency is denoted by *ω*, relaxation time is *τ*, ℏ is the reduced plank constant, and $\mu_{c}$ is the chemical potential of graphene. When the energy of a striking photon is higher (i.e., $\hbar \omega >2\left|\mu_{c}\right|$), the interband transition becomes effective for optical conductivity at higher frequencies [62]. In contrast to this, the electrostatic doping intensifies the electron carrier concentration in graphene. The photon energy becomes less than $2\left|\mu_{c}\right|$, at which point the intraband transition blocks the conductivity and interband transitions due to Pauli blocking. There are two common approaches used by computational electromagnetics calculations to hypothetically model graphene. One method is to utilize the effective bulk permittivity [63]. In-plane permittivity can be described by surface conductivity and the graphene layer effective thickness by relation as $\varepsilon_{r}=i\sigma^{s}/\left(\omega \varepsilon_{o}d\right)$, where *d* and $\sigma^{s}$ represent the thickness and surface conductivity of the graphene sheet, respectively. In addition, the out-plane permittivity should be considered as dielectric permittivity with a value of 2.5 according to [64]. The other method straightforwardly uses the surface conductivity by applying the condition of surface impedance [65,66,67]. Nevertheless, the conductivity of graphene attains diverse behavior at terahertz, infrared and visible frequencies by having negative real permittivity in bulk material due to kinetic inductance of electrons.

### 2.1. Surface Plasmon Resonance and Gate Tuning of Graphene Conductivity

Compared to noble metals, graphene supports the generation and travel of SPPs at the frequencies mentioned above. In particular, a free space standing graphene sheet supporting transverse magnetic (TM) plasmon mode can be calculated as:

(14) $$k_{spp}=k_{o}\sqrt{1-{\left(\frac{2}{\eta \sigma }\right)}^{2}}$$

(15) $$Z_{c}=\frac{k_{SPP}}{\omega \varepsilon_{o}\varepsilon_{eff}}$$

**Figure 2:**
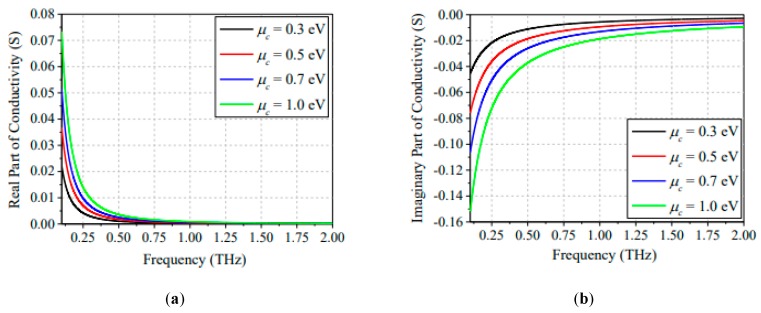
(**a**) Changing chemical potential has a significant effect on the real and imaginary parts of graphene conductivity at terahertz frequencies; and (**b**) conductivity behavior of graphene with respect to electrostatic biasing, i.e., changing chemical potential at higher frequencies. © 2019 APS. Reprinted with permission from [69].

Equations (14) and (15) can be used as an approximation for calculating the loss and leading wavelength of plasmons in innovative designs, such as graphene patches and graphene nanoribbons. *k_spp_* is surface plasmons wave vector and *Zc* is the characteristic impedance of the graphene sheet Undeniably, a standout among the most exciting properties of graphene is the ability to control the complex conductivity, which results in frequency tunability. This should be effortless by applying an external gate (electrostatic) bias to manipulate the graphene field-effect [68], when the voltage is applied vertically to the graphene sample [69]. The external bias applied onto the graphene injects more electrons and holes, consequently altering the chemical potential of graphene and resulting in frequency tunability, as depicted in Figure 2.

Typically, the gating of the graphene sheet controls the chemical potential of graphene by varying the bias voltage across the graphene sheet. The change in chemical potential by gate voltage tunes the graphene conductivity for the desired requirements. In addition, the variation of chemical potential enables the frequency tunability feature because of carrier injection, which increases the graphene conductivity; however, the chemical potential is electrically controlled by varying bias voltage on the graphene sheet. The relation between chemical potential and the bias voltage is derived through Equation (16). Ongoing examinations have affirmed that this tunability is exceptional in a broad range and quick up to some hundreds of gigahertz, thus empowering reconfiguration abilities in all graphene antennas. In a related context, magneto-statically biasing the graphene can produce a discontinuous band structure and become discretized in Landau levels. This behavior of conductivity can be exploited as a gyro tropic tensor, which leads to non-reciprocal responses [70,71,72]. In addition, the graphene conductivity and other properties can be modified by optical pumping, which can be used for terahertz lasing and reconfigurability purposes. All the above-mentioned phenomena can be considered for designing optical devices using graphene.

(16) $$Vg=\left[\frac{q_{e}\mu_{c}^{2}h}{\pi h^{2}v_{f}^{2}\varepsilon_{0}\varepsilon_{r}}\right]$$

*Vg* is the gate potential applied at gate pads of the graphene-based device and *μ_c_* is the chemical potential. The increase in the gate voltage actively tunes the graphene conductivity, which provides frequency reconfigurability of the device without changing the geometric dimensions.

### 2.2. Conductivity of Multi-Layer Stacked Graphene Sheets

It is worth mentioning that carbon-based nanomaterial composites have to overcome certain shortcomings, for instance low conductivity and toughness [73]. Additionally, single-layer graphene has relatively low conductivity and is practically hard to obtain higher conductivity in the zero-bias state. A possible solution to increase the conductivity of graphene is by stacking graphene layers. To calculate the conductivity of multi-layer graphene, the authors of [74] assumed that three layers of graphene are closely situated inside the stack and the effect of the dielectric substrate is strictly removed. The approach of electrostatic gate bias [74] is being applied to investigate the properties of graphene stack [75], which regulates the carrier density in graphene layers, and, afterwards, electromagnetic (EM) behavior of the graphene stack at the infrared region is analyzed. The proposed method is employed in two dependent stages. In the first stage, the carrier density is determined with respect to the applied gate voltage using an electrostatic bias approach [76]. In the second stage, the information obtained in the first stage is used to calculate the complex frequency-dependent conductivity of the stack. The carrier densities of tri-layer graphene biased with gate voltage can be approximated as

(17) $$qn_{s}^{top}=qn_{si}^{top}-C_{ox}^{top}V_{g}$$

(18) $$qn_{s}^{mid}=qn_{si}^{mid}+C_{ox}^{top}V_{g}+C_{ox}^{bot}V_{g}-C_{ox}^{mid}V_{g}$$

(19) $$qn_{s}^{bot}=qn_{si}^{bot}+C_{ox}^{top}V_{g}+C_{ox}^{mid}V_{g}-C_{ox}^{bot}V_{g}$$

where $n_{s}^{t,m,b}$ denotes the total carrier density of the graphene layers (*t, m* and *b* indicate top, middle, and bottom layers, respectively), $n_{si}^{t,m,b}$ characterizes the pre-doping of graphene layers and $C_{ox}^{t,\;m\;b}$ is the capacitance of the dielectric layer. When the values of carrier densities are known, the conductivity and chemical potential can be easily calculated to determine the EM behavior of the graphene stack [77]. Remember that this electrostatic method estimates the graphene sheets as an infinite ideal conductor to calculate the carrier densities. Merging Equations (14)–(16), the chemical potential of each graphene layer can be found through [78]

(20) $$n_{s}=\frac{2}{\pi \hbar^{2}v_{F}^{2}}\int_{0}^{\infty }\varepsilon [fd(\varepsilon -\mu_{c})-fd(\varepsilon +\mu_{c})]d\varepsilon$$

Once the Fermi energies (*µ_c_*) are recognized for each of the graphene sheets, the complex conductivity of the graphene stack–metal hybrid structure can be extracted as

(21) $$\sigma_{s}(w)=i\sigma_{o}\frac{4E_{f,s}}{\pi }\frac{1}{\omega +i\gamma }$$

Furthermore, the calculation of graphene conductivity for single and multilayer graphene up to three layers has a high sheet resistance and low electrical conductivity, especially when graphene is utilized in electronic and photonic devices. This could significantly bound the graphene application in bio-sensing, wireless sensors and imaging applications. Various studies have reported high electrical conductivities of multilayer graphene using different methods; for example, multilayer graphene is reported [79] to have high electrical conductivity and low sheet resistance by treating the graphene film at high temperature (2000 °C). The annealing process of the graphene film attains the electrical conductivity of 1.57 × 10^5^ S/m after being filtered from graphite nanoplatelets at high temperatures [80]. In another study [81], the ball-milling exfoliation technique for graphene is utilized during high-temperature annealing and acquired 2.2 × 10^5^ S/m for multilayer graphene film.

Most recently, the improved conductivity of graphene-based films is reported in [82], where the authors investigated the fragile mechanical toughness and low conductivity of graphene-based films, which serve as significant constraints for the use of multilayer graphene in electronic devices. The higher electrical conductivity of 1.1 × 10^6^ S/m is acquired with a carrier mobility of 808.8 cm^2^/Vs. This high conductivity is achieved by slowly heating the polyimide precursor to 1300 °C to form the amorphous carbon structure and eliminates the non-carbon atoms. Finally, the electrical conductivity of multilayer graphene film is improved by increasing the temperature of 2850 °C. Tang et al. experimentally demonstrated multilayer graphene with a high electrical conductivity of 10^6^ S/m [73]. The graphene films exhibited a high tensile bending of 9.36 and a strain sensitivity of 9.8, which makes them preferable for wearable flexible antennas for sensing applications. In a related study [83], highly conductive graphene multilayer films are utilized in flexible filters. The high conductivity of graphene layers improves the performance of the filter. In addition, the filter remains stable in operation after being bent 200 times with no mechanical failure.

### 2.3. Electromagnetic Interaction and Computational Modeling of Graphene Antenna Devices

The interaction of graphene with electromagnetic waves has been an intensive research subject in recent decades. Investigations of graphene electromagnetic properties first considered solving Maxwell equations to simulate EM wave propagation along the graphene surface [84]. However, in certain scenarios, Maxwell equations lack analytical methods except for a few simple recognized problems. Thus, numerical techniques are essential to comprehend guiding wave propagation and scattering of graphene [66]. Numerous computational electromagnetic methods are employed for the simulation of graphene-based antenna devices, among which the most popular methods are: finite difference time domain method (FDTD), method of moments (MoM), and finite element method (FEM). Every numerical technique has its own merits and demerits depending on the type of the particular problem. Various commercial simulation software packages based on the above numerical techniques are available for the modeling of graphene and graphene-based devices (e.g., CST, COMSOL, HFSS, FEKO and FDTD Solutions) [66].

The FDTD method has a specific advantage in problems involving transient and broadband problems, where broadband results can be obtained in one simulation. Compared to the frequency time-domain numerical method, it requires minimum computing memory and less simulation time. Moreover, FDTD is based on an iterative scheme, which can solve large linear systems, making it a simple and robust method for graphene-related simulations. Nayyri et al. investigated the EM interaction by modeling graphene using FDTD with surface boundary conditions. The graphene is implemented as a conducting surface boundary and the dispersive conductivity of graphene is calculated through existing analytical methods [65]. In a related study, the author of [85] developed a FDTD method with finer spatial grids, allowing discretized steps in space and time. The approach of subcell FDTD can be employed for the modeling of the electrical thin graphene sheet [86], where graphene is considered as a thin layer. However, the subcell FDTD method lacks the modeling of infinitely thin graphene sheets and requires a certain type of perfectly matched layer. In another study [87], the formulation for graphene FDTD modeling is investigated based on the piecewise linear recursive convolution with thin material sheets techniques. The authors modeled the thin graphene sheets by specific recursive equations obtained from normal electric field components, thereby allowing the gating of the graphene sheets with bias voltage. Additionally, the conductivity of the graphene sheet is derived in Yee’s three-dimensional lattice for the FDTD simulations. In recent work [88], a novel method of modeling magnetized graphene in FDTD domain is demonstrated by using an anisotropic surface boundary condition. The physics behind these effective techniques is to incorporate magnetically-biased graphene into the EM wave scattering problem at the macroscopic scale to replace the graphene anisotropic conducting sheet. In addition, the surface conductivity matrix of magnetized graphene is applied for the infinitesimally thin graphene sheet. The localized surface plasmon resonance in the graphene-based sandwich structure is demonstrated using the FDTD simulation and provides the strong interaction of graphene with metals having higher EM wave confinement [89].

The finite element method is the full-wave computational method for problems containing electromagnetic boundaries. The essential principle of FEM is to discretize the entire computing domain with subdomains of finite number, where the unknown functions are extended by straightforward interpolation with unknown coefficients [66]. Then, a set of algebraic equations of the unknown coefficients are obtained utilizing Ritz variational or Galerkin’s method. Lastly, by solving this as a linear system, the solution for the whole domain can be calculated. FEM also has an advantage in complex and inhomogeneous problems, where the triangular or tetrahedral elements are applied for arbitrary geometries accurately. Brar et al. [90] modeled graphene by solving Maxwell’s equation with the FEM method using the graphene sheet thickness of 0.1 nm and demonstrated an increase of light–graphene interaction at the infrared frequencies. The FEM analysis of reconfigurable antenna illustrates that the impedance of the antenna can be tuned by increasing the chemical potential of a multilayer graphene stack [91]. A new computational method known as discontinuous Galerkin time-domain (DGTD) has extensively contributed to the study of graphene sheet interaction with EM waves. The DGTD method is included in the Lumerical software, where analysis regarding optical and surface heat losses can be simulated. DGTD is very efficient for problems involving multiscale structures, allowing the decomposition of the domain into several subdomains and discretizing each subdomain separately. The computational operations in DGTD are local, thus a bigger matrix can be divided into smaller matrices. Li et al. [92] conducted a simulation of a thin graphene sheet with the DGTD method to analyze the transient response of graphene ranging from microwaves to terahertz frequencies and obtained the resistive boundary conditions by considering an infinite graphene sheet with finite surface conductivity [93]. The approach based on DGTD-WE method [94] has been proposed for the analysis of graphene-based devices, where numerical fluxes related to the transmission boundary condition are utilized. Additionally, a vector fitting technique is employed to calculate the surface conductivity of a thin graphene sheet in terms of series partial fractions.

In contrast to the above-discussed computational methods, which solve the EM problems through differential equations, method of moments (MoM) is a computational technique employed for solving electromagnetic sources as surface or volume integral calculations in the frequency domain. The advantage of MoM is that it only considers the EM sources where the surface or volume current on the surface of geometry needs to be discretized. Moreover, MoM is exceptionally effective for electrically large electronic devices and is widely utilized for calculating radiation and scattering properties by deriving surface currents through Green’s function. The analytical equations based on dyadic Green’s function are derived for graphene in [95]. Araneo et al. performed the simulation of graphene nanoribbons by applying the MoM technique to solve the relevant electric field integral equation [96]. The authors also expended the spatial domain formulation to treat the weak nonlocal conductivity of graphene nanoribbon via an original integrodifferential approach by considering the graphene spectral conductivity model with low wavenumbers. In another work, graphene nanoribbons are studied by space-domain MoM [97]. The spectral representation of graphene is approximate using Bhatnagar–Gross–Krook approximation on the semiclassical framework. The calculation obtained through this method can allow the effective treatment of multilayer graphene nanoribbon with spatially varying carrier density. Furthermore, the investigation of graphene-based microstrip structures is studied through spectral domain and the method of lines with the conductivity of graphene being modeled through the tensor matrix [98]. Hatefi et al. also validated the two numerical methods by simulation of the graphene-based patches in COMSOL.

An effective way to validate the computational results of graphene-based devices is by designing the equivalent resonant circuits and conventional transmission line (TL) models for graphene-based antenna simulations. Lovat demonstrated the EM interaction of graphene by deriving equivalent circuits as the EM wave propagates through the graphene sheet [29]. The effect of electric and magnetic bias is established through the equivalent circuit to study the shielding properties of graphene sheets against striking EM plane waves, which provide an easy method for the design and analysis of graphene structures with tunable graphene conductivity. The equivalent circuit per unit length is introduced in [62], where the author calculated the analytical expression for the graphene conductivity and circuit lumped components and illustrated the propagation of surface waves along spatial graphene waveguides. Recently, the equivalent resonant circuit model was designed by Zhang et al. for the validation of simulated results, and extracted resistive, inductive, and capacitive (RLC) parameters by considering the graphene bowtie antenna as one port resonator [78]. Additionally, the partial element equivalent circuit (PEEC) method is utilized for designing the equivalent circuit of the graphene-based terahertz antenna [99]. The graphene in the PEEC model is replaced by the impedance boundary condition and the surface conductivity is transformed into a vivid circuit for the reduction of simulation time. In the related context, the analysis of graphene antenna is simulated by adopting transmission line model for predicting the efficiency of reconfigurable dipoles [100], while the properties of magnetically biased graphene-based nonreciprocal spatial isolator are studied through equivalent dyadic transmission line model to review gyrotropic properties of graphene [58]. Lastly, the input impedance and efficiency of the plasmonic graphene antenna are exploited through the theory of the characteristic modes [101].

## 3. Graphene Resonant Terahertz Antennas

**Figure 3:**
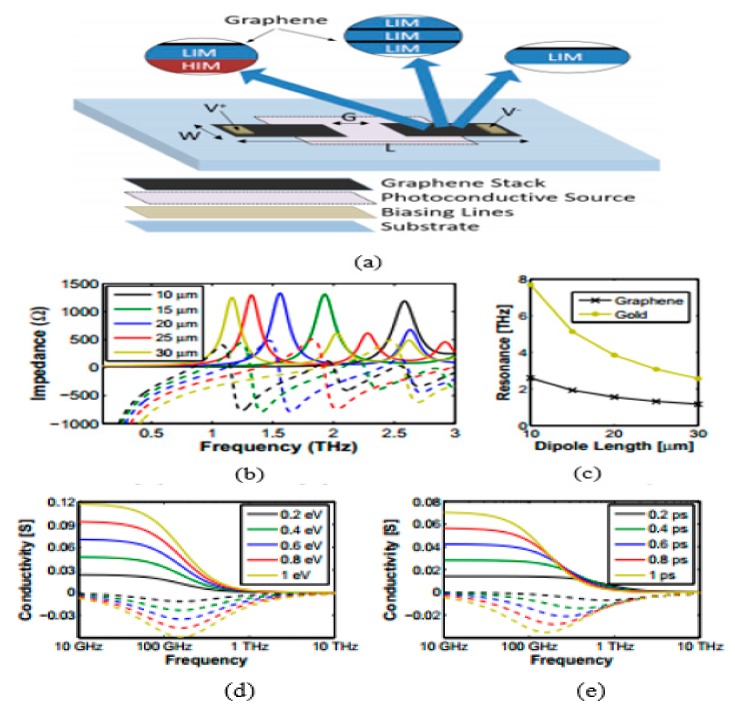
(**a**) Graphene stack dipole antenna on low index material (LIM); (**b**) input impedance and resonance frequency as a function of dipole arm length, where the frequency shifts to lower end as the length increases; (**c**) comparison of graphene radiator with gold, as gold radiator resonates at a higher frequency than graphene; and (**d**,**e**) behavior of conductivity with respect to changes in chemical potential and relaxation time. © IEEE 2017, Reprinted with permission from [102].

Nanoantennas operating in THz band are of great importance in everyday life due to their numerous utilizations in the various photonics fields. Initially, graphene-based antennas were hypothetically studied, while recent studies by Sergai and colleagues [102] demonstrated graphene as the radiating element in a stack graphene-based dipole antenna that resonates at terahertz frequency band, as depicted in Figure 3a. The dipole antenna is designed by employing graphene sheet stacks on low index material (LIM; i.e., quartz) substrate with the dielectric constant $\varepsilon_{r}$ = 3.8. It has been found that the metallic counterpart of the graphene antenna resonates at somewhat higher frequencies than the graphene antenna. By increasing the length, L, of the dipoles and adjusting the gap between the two dipole arms, the antenna can resonate at lower frequencies, as can be observed in Figure 3b. By keeping the relaxation time at τ = 1 ps and chemical potential at $\mu_{c}$ = 0.2 eV, the radiation patterns at the resonance frequency remain stable, while significant improvement can be seen in radiation efficiency because of graphene as the radiator.

**Figure 4:**
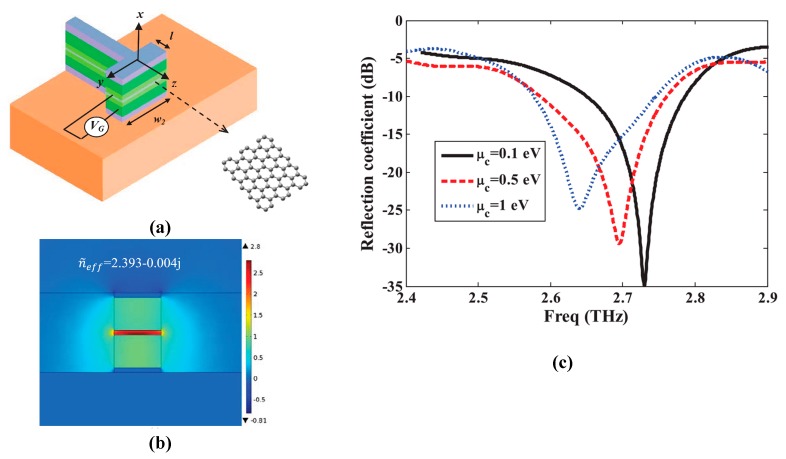
(**a**) Graphene stack antenna with waveguide graphene–metal hybrid structure fed, where the gate voltage is applied to graphene layers and the antenna substrate is quartz with silicon stack; (**b**) effective refractive index mode of graphene–metal waveguide; and (**c**) reflection coefficient of the proposed antenna as a function of chemical potential, where, as the chemical potential is increased to $\mu_{c}$ = 1 eV, the resonance frequency becomes lower side. © Springer 2019. Reprinted with permission from [103].

Hossein et al. proposed a graphene–metal hybrid antenna by employing waveguide feed [103], as depicted in Figure 4a. The authors employed the graphene–metal waveguide structure, which can support TEM mode, thus the transmission line model is adopted for feeding the antenna through a waveguide. The proposed antenna resonates at 2.7 THz; to achieve tunability over the broad range of frequency, few-layer graphene sheets are used. The gate voltage is applied across the graphene sheet by keeping the dimensions and refractive index of waveguide constant, and the resonant frequency is tuned to 2.63 THz, as illustrated in Figure 4c. It is worth noting that increasing chemical potential through gating the single-layer graphene can result in a small change in frequency shift relative to multi-layer graphene. Rajni et al. [104] studied the effect of appropriate material on the graphene patch antenna. Various materials have different performances due to their dielectric permittivity, which affects the graphene patch antenna characteristics; to achieve higher bandwidth and radiation efficiency, thicker substrates are preferable. The graphene model in this work is assumed to portray zero chemical potential, which limits the radiation efficiency. However, the radiation efficiency can be increased by using a substrate material with greater dielectric constant. In another study, a graphene-based plasmonic nanoantenna at terahertz band for terahertz communication is designed and analyzed [105]. Coupling theory is utilized to overcome the limitation of low radiation. Arrays of the proposed antenna are electromagnetically simulated to achieve a high total gain while keeping the size compact. Modeling of mutual coupling between antenna elements is done by various approaches such as the cavity model, transmission line model and method of moments. These models play an active role in conventional metallic antenna arrays but have many drawbacks for plasmonic nanoantenna arrays. One of the key drawbacks is simplifying the model by neglecting the surface waves, which is a very critical element for the resonance of antennas at terahertz frequencies.

**Figure 5:**
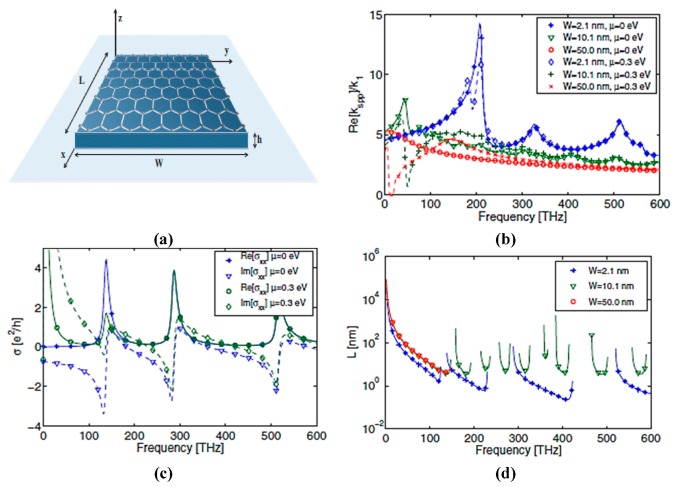
(**a**) Graphene nanoribbon (GNR) antenna; (**b**) real part k_spp_/k_1_ surface plasmon with increasing width of GNR; (**c**) real and imaginary parts of graphene nanoribbon conductivity by keeping the width of patch constant W = 2.1 nm, where the blue line shows the conductivity behavior of GNR at $\mu_{c}$ = 0 eV and the green line depicts the conductivity at the chemical potential of $\mu_{c}$ = 0.3 eV, by increasing the chemical potential the conductivity shifts to higher frequencies; and (**d**) nanoantenna resonant length L at different widths of GNR with $\mu_{c}$ = 0.3 eV for the transverse magnetic (TM) mode. © Elsevier. Reprinted with permission from [33].

The use of graphene nanoribbons (GNRs) in a dipole antenna is presented in [106]. The authors considered the extinction cross-section of the antenna structure to determine the resonating frequency; however, they did not deliberate on the direct excitation of surface plasmon polaritons (SPPs) to study the transmission. The antenna is designed by transferring graphene ribbons onto the dielectric material. Graphene patches and ribbons can operate and resonate at THz frequencies; these phenomena arise due to the high kinetic conductance of graphene, which excites SPPs of short wavelength in the terahertz frequency range [107,108]. The frequency of operation is determined by SPP propagation across antenna length. The absorption and extinction cross-section of the designed antenna can be easily obtained by the effective polarizability of the nano-patch when resonance frequencies are available. In another study [33], the same authors exploited the performance of SPPs wave in graphene nanoribbons of semi-finite size, as presented in Figure 5a. For the lateral confinement of electrons and their effect on the conductivity, graphene nanoribbons are analytically studied through the Kubo formula, as illustrated in Figure 5b. The nanoantenna proposed in [106] is designed as a plasmonic resonant cavity. By exploiting SPP wave in GNRs and considering the high mode compression factor, a graphene-based plasmonic antenna operating at a much lower frequency compared to metallic antennas, with varying chemical potential as well as antenna length, can be reconfigured to various frequencies, as shown in Figure 5c,d. Tamagnone and coworkers proposed a graphene dipole antenna and studied the gap between far-field radiation and propagating surface plasmons on the graphene sheet [109]. As part of their contribution to this field, they developed a port between the dipole arms, which allows them to excite surface plasmons on the graphene sheet, and studied the input impedance and efficiency of the GNR antenna. Moreover, electrostatic biasing of the graphene sheet in the antenna and selection of an appropriate value for the extraordinary tunability of THz sources are verified.

More work on the graphene patch antenna has been done by the same researchers. To increase the directivity of a graphene patch antenna, the double-layer graphene stack and the dielectric lens are utilized. These methods can be directly employed in applications that require high directivity due to high tunability over a wide range. Furthermore, resonant tuning of frequency is established by having impedance reliability, and it is noticed that the efficiency alters the frequency reconfigurability and is not stable, similar to input impedance, which can be explained through the electrical length of the antenna. The plasmon wavelength is increased by varying the chemical potential of graphene layers through biasing, which produces upshift in the resonance frequency, thus increasing the electrical length of the antenna at the same physical size. This standard method is employed for miniaturizing graphene antenna devices, which exploit high inductance of graphene for low chemical potential with the cost of reduced radiation efficiency. Using high chemical potential, the SPP properties of graphene are rarely affected by the bias voltage; thus, the reconfigurability of the graphene antenna is hardly affected.

**Figure 6:**
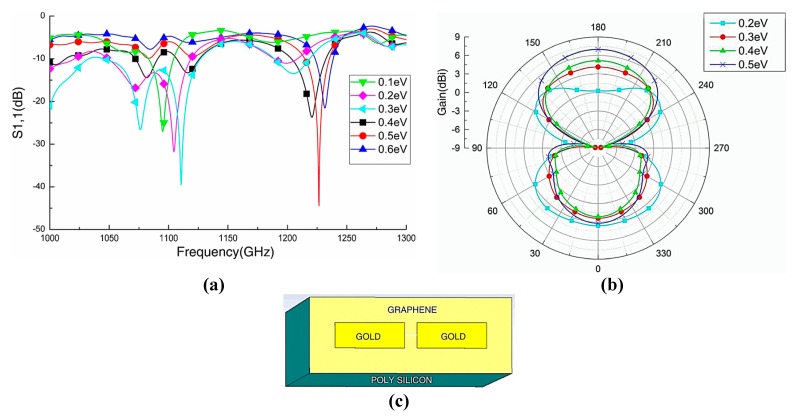
(**a**) Schematic of the graphene–metal hybrid antenna, where the graphene layer is inserted between quartz substrate and gold patches; (**b**) reflection coefficient hybrid antenna with respect to chemical potential; and (**c**) gain polar plots and changes in bandwidth by increasing the chemical potential. © 2016 IEEE. Reprinted from [110].

**Figure 7:**
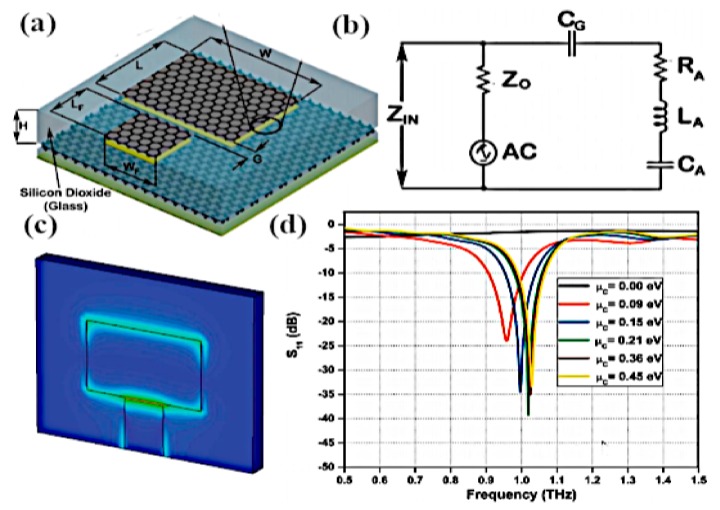
(**a**) Configuration of the coupled fed graphene antenna designed on a glass substrate; (**b**) RLC resonant equivalent circuit model for graphene antenna; (**c**) propagation of SPP wave on antenna surface; and (**d**) improvement in return loss by increasing the chemical potential of graphene through gate biasing. © 2018 Wiley, Reprinted with permission from [112].

Qin et al. worked on the graphene–metal hybrid antenna designed on a SiO_2_/Si stack substrate by sandwiching the graphene layer between the metal and the substrate [110]. This technique is used to increase the gain of a nanoantenna and to control the conductivity of graphene by applying the gate voltage, which produces shifts in frequency by increasing the chemical potential. The thickness of Si is t_1_ = 260 µm and the thickness of silica is t_2_ = 300 nm, while gold metal is employed as a radiator for the dipole, as illustrated in Figure 6c. By positioning the graphene layer as a parasitic element close enough to the dipole, a resonant shift in operating frequency is observed from 1.095 THz to 1.232 THz; in addition, tunability over frequency is realized by increasing chemical potential through biasing voltage. It is worth noting that, by using graphene as a high resistance sheet, the antenna moves towards matching conditions due to its good reflection coefficient, as can be seen in Figure 6a. In Figure 6b, it can be observed that increasing the chemical potential of the graphene layer has a significant effect on the gain of the antenna. By increasing the chemical potential value from 0.2 to 0.5 eV, the beam width along with the gain of the antenna is increased. Reefat and coworkers presented a graphene dipole antenna designed on a stacked substrate as well as its chemical potential and relaxation time constant [111]. The gap between dipole arms is used to excite the antenna, which allows the graphene strips to propagate surface plasmons at terahertz frequencies. It is observed that, by using quartz/silicon substrate, the antenna performs better in terms of radiation efficiency and gain. The authors increased the absorption cross-section of the antenna by increasing the size of the substrate, which further enhances the characteristics of graphene antenna. In another work, Goyal et al. investigated a graphene nano-patch with a metal-polished quartz substrate to achieve the high gain and better radiation efficiency required for terahertz communications [112]. The graphene patch antenna is designed on a glass substrate and the radiation characteristics of the antenna are tuned by exploiting graphene surface impedance, as presented in Figure 7a. By integrating graphene with metallic radiator and increasing the chemical potential of graphene patch, the performance of the antenna is improved (Figure 7d). The radiative graphene patch is couple-fed through the transmission line and the antenna is simulated by the full-wave solver. The graphene sheet edges on the dielectric substrate are assumed to be a mirror, and the graphene patch is the main resonator for SPP wave. Furthermore, a circuit model is developed to calculate the resonance condition, as this model was first used for the calculation of surface impedance of high conducting radiator. The circuit model, as illustrated in Figure 7b, inserts the capacitance between the transmission line and graphene patch along with t reactance at the edges of patch to minimize the effect of fringing fields. By incorporating these circuit elements, resonance of antenna and radiation efficiency can be directly calculated through transmission line theory. The edges of antenna produce maximum radiation, which can be observed in Figure 7c.

The idea of graphene-coated wires (GCWs) was recently presented by Diego and colleagues [113] to further enhance the radiation efficiency of graphene antennas. The dispersion relation is diagnostically developed by the authors for the variation of tabular waveguide designed supporting both electrical and magnetic biasing and demonstrated that SPP propagation substantially decreases in rolled-up structure relative to the designs, which are strip based.

**Figure 8:**
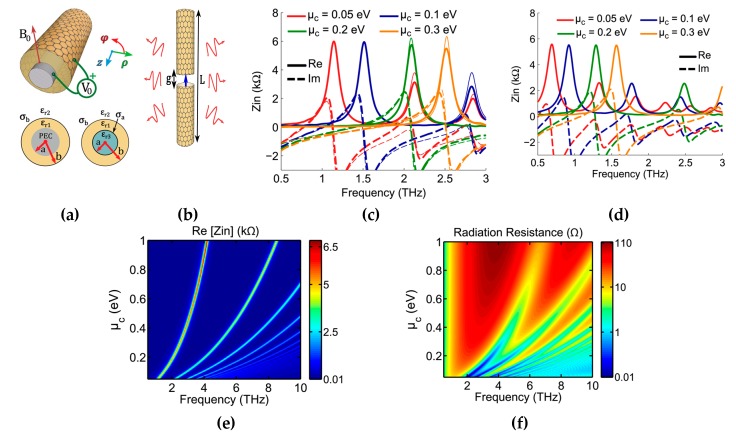
(**a**) Configuration of graphene-coated wire (GCW); (**b**) geometry of dipole arms of the GCW waveguide structure, where gap “g” is used to excite the GCW and acts as the capacitance between the GCW; (**c**) input impedance of double-layer GCW as a function of chemical potential, where a broad shift in frequency is observed by increasing the chemical potential to 0.3 eV; (**d**) input impedance comparison of the double-layer planar graphene antenna structure, where it is clearly observed that tunability over the range of frequency can be better achieved through GCW concerning the graphene planar structure, as a smaller shift occurs over frequency when chemical potential is increased; and (**e**,**f**) radiation resistance with chemical potential. © 2015 IEEE. Reprinted with permission from [113].

Additionally, all power propagates outside the wire material; hence, these type of waveguide cylinders are designed as a resistant for the dielectric losses in the material [114,115,116,117]. These discoveries have significant difficulties in the optimum design of ideal THz communication interlocks and reconfigurable waveguide gadgets, particularly for resonant antenna designs. The graphene patch antenna has the benefit of planarity; however, in terms of radiation efficiency, GCWs outperform planar graphene antennas for the same configuration and materials. The GCW dipole antenna has improved performance in terms of robustness to dielectric media losses, SPP quality factor and trapping of optical energy by the appropriate substrate. Figure 8f presents minima and maxima of radiation, which correlate to even and odd multiples of resonating plasmon wavelengths at dipole arms. The subwavelength current influence cancels the effect of far-field by having radiation nulls and highly confined surface plasmon.

Furthermore, the authors proposed an equivalent circuit model, which assumes the capacitance between the gap of GCW arms and reactance at the edges of a dipole. The geometry presented in Figure 8a,b for GCWs has a perfect electric conductor as a core of the wire to enable surface plasmon propagation. The condition states that there must be different real signs of dielectric permittivity at the interface. Graphene is wrapped on a cylindrical glass substrate, as it acts as a conductor due to the negative real part of permittivity at terahertz frequencies. This property of graphene allows the propagation of surface plasmons on GCW dipole arms [110,118]. Increasing the chemical potential of rounded graphene around glass substrate results in the propagation of surface plasmon at higher frequencies, as depicted in Figure 8c. In Section 3, the essential graphene-based nanoantennas are reviewed on the basis of resonance frequency; however, numerous researchers worldwide have sought specific approaches to improve the performance further and accomplish better characteristics of graphene nanoantennas [119,120]. The advancement done in the field of graphene antennas is too abundant to study in detail here, including ideas for enhancing the radiation efficiency and integration of graphene antenna with photomixers [121].

**Figure 9:**
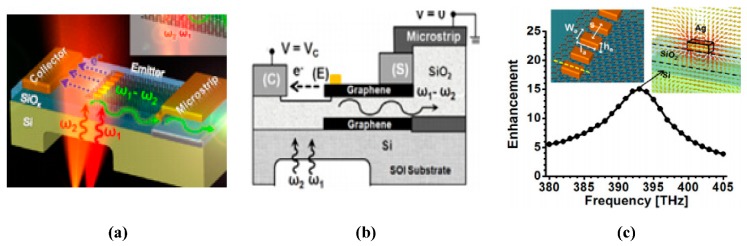
(**a**) Graphene optical antenna model excited by laser beams; (**b**) cross-sectional view of graphene patches integrated with photomixer; (**c**) enhancement of averaged optical field versus frequency, where the silver antenna array on the graphene sheet has a better enhancement factor and electric field distribution at the point of contact. © 2013 IOP. Reprinted with permission from [122].

Chen et al. demonstrated the concept of terahertz frequency generation by using a graphene-based antenna along with photomixer to achieve wide-band and tunable performance over the terahertz region [122], as presented in Figure 9a,b. By optical heterodyne, terahertz currents are produced at the radiator tips, when the laser beam is focused on graphene nano radiator. A plasmonic antenna is used in [100] to manipulate the optical transparency of graphene, thereby boosting the overall photocurrent generated through the mixing of laser radiation and graphene nanoantennas, as illustrated in Figure 9c. The most important hurdle in THz communications is providing a wide frequency bandwidth for operation and large power generation, which can be overcome by the efficient coupling of laser emissions with graphene antennas. Electron field emission, having ballistic transport in space, has the further benefit of insensitivity to environment temperature and ionizing radiation.

Consequently, field outflow in nanodevices with rapid switching time and high current densities are generally exploited in miniaturized nanoelectronics [123]. Mario and colleagues worked on the idea of different shape tactics to enhance graphene plasmons propagation [124]. Intrinsic losses and defects in the substrate have a negative effect on graphene plasmons. Thereby, graphene plasmons have low propagating length and short periods of decay. A feasible method to launch plasmons in graphene waveguide structure is employing noble metal antennas, which face major challenges for effective coupling and propagation of plasmon viable for on-chip communication. The authors analyzed several nanoantenna designs with numerical simulations and coupling of metal plasmons to graphene plasmons, as well as studied the effect of different graphene waveguide structure along with metal antennas for the efficient propagation of graphene plasmons [125]. Moreover, in comparison with a dipole antenna, the Yagi–Uda antenna [103] performs better with stronger coupling with graphene plasmons, which allows for more directive propagation. Strong plasmons are activated by dipole antenna in tapered graphene waveguides by the constructive interference of plasmon reflections, generated at the edges of the design structure. The directivity of propagating graphene plasmons is significantly boosted by placing chains of graphene fractal triangles in waveguides [126,127].

**Figure 10:**
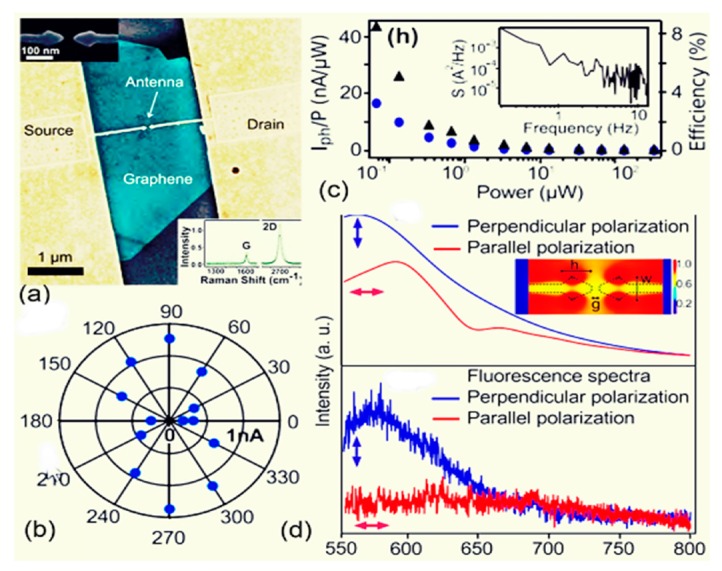
(**a**) Design of graphene antenna photodetector device connected with external electrodes; (**b**) 2D polar plot showing the magnitude of photocurrent and polarization isotropy of photocurrent generated from device; (**c**) external quantum efficiency and photoresponse with respect to power depicting the linear response for biasing; and (**d**) finite difference time domain (FDTD) spectra from simulation and spectra of fluorescence, which shows the polarization dependence on the luminescence of gold antenna. © 2014 AIP Publishing. Reprinted with permission from [128].

Moving toward the infrared (IR) and visible frequencies, researchers widely investigate the effect of graphene antenna responses on photodetection and light enhancement by the manipulation of localized surface plasmons. Chitraleema and coworkers investigated the performance of graphene-metal antennas for enhanced photodetection [128]. A four orders of magnitude performance enhancement is achieved when no gate voltage is applied to graphene layers relative to a simple metal antenna. It is observed that near field enhancement is low, due to the excitation of off-plasmon resonance, although the field enhancement is better around the antenna edges. As the source beams become parallelly polarized to the antenna axis, the resonance is suppressed because of the design of the antenna with an unusual polarization response, as depicted in Figure 10d. The main motivation was to mitigate the effects of resonance damping, which comes from the fingers connecting the graphene antenna to electrodes, as clearly observed in Figure 10b. Under zero bias voltage, the quantum efficiency is low, resulting from the 2.3% absorption of graphene, but the responsivity is increased as the gate voltage is applied, while the light-harvesting efficiency of the photodetector is increased by up to four orders of magnitude with respect to metallic antennas, as illustrated in Figure 10c. Figure 10a depicts the schematics of a graphene antenna integrated into a photodetector.

**Figure 11:**
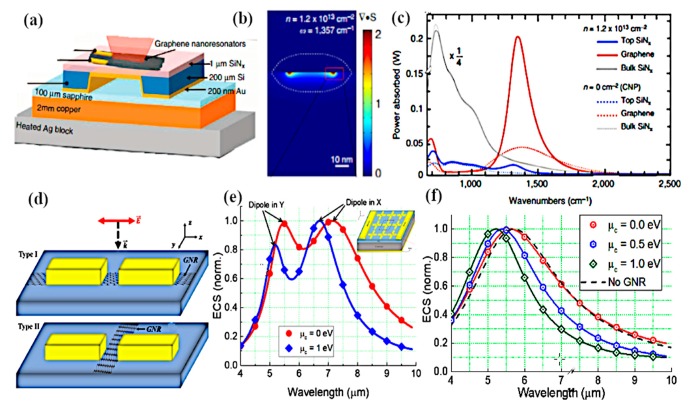
(**a**) Geometry of graphene nano-resonators placed on a silicon nitride (SiN) substrate having a thickness of 1 µm and a back reflector of AU material of 200 nm thin membrane. The gate voltage is applied through SiN substrate and graphene sheet. Copper of 2 mm thickness is employed for temperature control base for the heating of silver layer, while a layer of sapphire is used for electrical isolation. (**b**) Finite element simulation of graphene nanoresonator for plasmon resonance, which has the carrier density of 1.2 × 10^13^ cm^−2^, by using 40 nm graphene resonator on 1-µm SiN with gold as a back reflector. (**c**) Power density and absorption comparison of a 40-nm graphene resonator on top of a bulk silicon nitride substrate. (**d**) Dipole antenna loaded with graphene: Type 1 has a graphene layer below the dipole arms and in horizontal position, while Type 2 has a graphene layer in vertical position and is placed between the dipole gap. (**e**) Normalized extinction cross-section (ECS) of graphene–metal antenna, where the blue line represents ECS of the dipole with longer arms at x-coordinate with chemical potential of 1 eV, while the red line is with respect to zero chemical potential. (**f**) Tuning of resonance frequency of Type 1 antenna with increasing chemical potential. Copyright © 2015 American Chemical Society and © 2018 IOP Publishing Ltd. Reprinted with permission from [129,133].

Brar et al. experimentally demonstrated blackbody emission from graphene resonator and the control of emission with electronic tunability of a graphene antenna placed on a silicon nitride substrate [129]. It is shown that the graphene resonators produce antenna-coupled blackbody radiations with thin spectral peaks in the infrared region, as presented in Figure 11b,c. The experimental setup and schematic of graphene nanoresonators are shown in Figure 11a. By consistently varying the carrier density in graphene resonator, the intensity and frequency of spectral lines can be modified through gate voltage. The authors also explained the non-radiative transfer progression between the graphene sheet and dielectric material (SiN). The control of radiation emission resulting from the combination of plasmon–phonon and electron–plasmon exchanges by electronic gating has also been reported. Furthermore, the resonant frequency of graphene plasmon varies by increasing the carrier density and width of the resonator. Adam and colleagues critically analyzed the generation of plasmons by graphene together with gold nanodisk resonators and studied the near-field interaction using femtosecond laser pump measurement [130]. They observed that graphene plasmon generated at infrared wavelengths with the aid of increasing carrier density is dominated by the transfer charge process from gold metal to graphene because of the direct excitation through optical fields. Nanodisks produce optical resonance similar to tunable dipole antennas, which can strongly couple the far-field due to the close packing of the array structure. In another work, a graphene loaded metallic antenna is employed for the optical modulation from infrared to visible wavelengths. The application of such hybrid graphene–metal antenna can be exploited in sensing and communication fields. The authors used the graphene–metal antenna approach, which operate on low power and provide fast switching in microelectronic devices, instead of using large photodetectors. The planar nanostructure with graphene sheets (hybrid nanostructures) can tolerate extremely tunable plasmons at the optical region. By exciting nanostructures through external incident light and tuning the resonance of graphene plasmons with gating, the reflection and absorption of incident light can be varied; thus, tunability from infrared to the optical region can be achieved [131]. Dipolar antenna with dual gating is described by the authors of [132] for the detection of terahertz radiation by exploiting the photoelectric effect. A gap of 100 nm is chosen between dipole arms, which makes the PN-junction between the graphene sheet and the top surface of a dipolar gold antenna have excellent photosensitivity by concentrating the incident radiation across the junction. The gap between dipole arms serves as focusing element for terahertz radiation and the confined fields of the dipolar antenna result in higher absorption and generation of hot carriers. Moreover, by patterning the graphene sheet in H-shape configuration and keeping the inside width of the H-shape structure narrow, an enhancement occurs in the photoresponse. By increasing the width of the H-shape graphene arms, the whole resistance of the photodetector is lessened by reducing the contact resistance between the graphene and metal electrodes.

The optical properties of graphene are reliant on the exclusive gate biasing, making graphene electrically tunable at optical frequencies. In recent work, Yuwei and coworkers investigated the polarization control of graphene–metal nanoantenna with electrostatic biasing of monolayer graphene [133]. The design idea employed for tunable polarizer by the authors is to use the asymmetric cross antenna, which is comprised of a dual dipole antenna, orthogonal to each other, with the same feed gap between the orthogonal dipole arms, as presented in Figure 11d. Incrementing the chemical potential of the graphene layer, which is placed beneath the cross resonator by electrostatic doping, the resonant wavelength of the cross-dipole antenna loaded with graphene is shifted in the mid-infrared region at 500 nm. The shortening of the dipole arms results in the lower shift of frequency because of the lower influence of graphene on the optical response of dipole antenna as compared with larger dipole arms. The polarization behavior from circular to linear is tuned by electrically tuning the reflected wave. This control of polarization and the efficient light–graphene interchange with metallic nanoantennas make it more applicable in electrically controllable high-speed optoelectronic devices. Graphene single layer, regardless of monoatomic thickness, has exhibited critical tuning of metallic nanoantenna response with the enhancement of interacting localized fields. Figure 11 (where (e) represents the normalized extinction cross-section (ECS) at two different values of chemical potential from 0 to 1 eV) clearly shows that resonance of the cross antenna shifts to a higher frequency when the chemical potential is set to 1 eV; in addition, by increasing the length of dipole arms at x-coordinates, the resonance peak is shifted towards lower frequencies. Sasmita and coworkers proposed the graphene patch nanoantenna on a stacked substrate with dynamic control of surface conductivity when an electric field is applied across graphene sheets [134]. They further investigated the effect of SiO_2_ substrate thickness on the propagation properties of SPP on the graphene surface and control of SPP through SiO_2_ thickness, when it is less than 100 nm. This study again exemplifies the tradeoff between compact size and wavelength response. Graphene has been used as a parasitic element in various research works, for example reconfigurable substrates [135] or high impedance surfaces [136,137]. The key graphene antennas discussed in this section are summarized in Table 1, and can be employed for high-speed terahertz communication, terahertz transmitters and on-chip communications for enormous data rates.

## 4. Tunable Hybrid Graphene–Metal Plasmonic Nanoantennas and Their Characteristics

The graphene layers can be N-type or P-type doped by applying positive or negative voltage bias. Tunable surface conductivity in a graphene sheet can be achieved by the displacement of Fermi energy level (chemical potential) from the Dirac points, which results from the infusion of hot carriers in the graphene sheet. Graphene sheets sustain metallic responses and can maintain strong plasmonic behaviors from the terahertz region to mid-infrared and even some portion of visible light frequency region, due to the forbidden interband transition by the Pauli blocking. Significantly, single graphene layer with tunable characteristic (i.e., chemical potential tuning by gate bias) empowers the design and manipulation of its surface conductivity and, afterwards, controls the graphene plasmons to tune the plasmonic responses of graphene-loaded nanoantennas. In this section, the reputed antennas with graphene layers as the fundamental element identified for tuning the radiation characteristics and acquiring tunability are discussed. In the following subsections, the features of graphene–metal antennas (i.e., absorption, modulation and plasmons utilization) are discussed with the aid of graphene tunable conductivity behavior.

Remarkable work was demonstrated by Chen et al., who designed a graphene–metal nanoantenna comprised of two symmetric gold strips deposited on two different graphene layers at mid-infrared frequencies [139]. This investigation illustrates that high directive radiation from the edge of the graphene–metal antenna is due to the localized surface plasmon resonances (LSPRs), resulting from the constructive and destructive interference of LSPR radiation emitting from the gold nanostrips. The two-point dipole model is adopted to analyze the characteristics theoretically and FDTD analysis is used to simulate the performance of nanoantennas. The study further elaborates that, when the reflective index of the substrate medium and environment is increased, the scattering directivity beam pattern changes linearly. This scattering direction of the beam pattern is dynamically tuned through tuning the LSPR of the gold strips and altering the chemical potential of the parted graphene layers.

**Figure 12:**
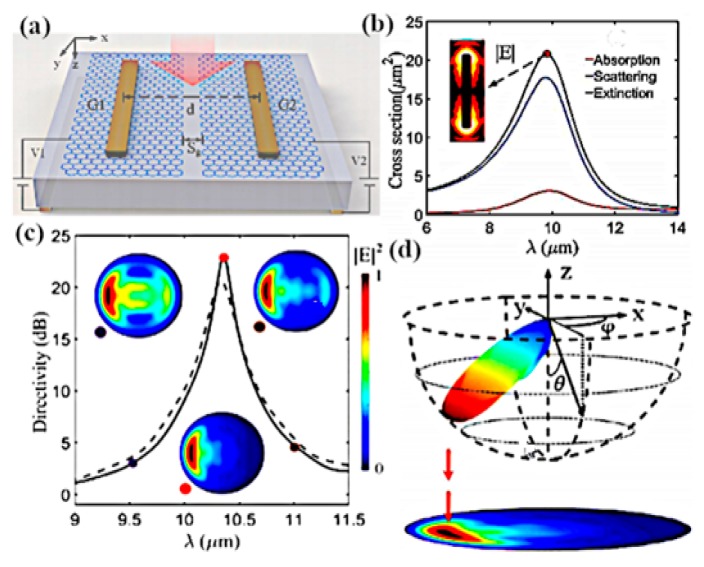
(**a**) Geometry of the graphene–metal nanostrip plasmonic antenna with the graphene placed under the nanostrips and two more strips beneath the quartz substrate where the gate voltage is applied to both graphene sheet. (**b**) Simulated extinction, absorption and scattering responses of the proposed antenna with a single gold nanostrip keeping the Fermi energy of the graphene sheet *E_f_* = 0.8 eV. The electric field distribution at the resonance peak shows that the electric field strength is greater at either side of the gold nanostrip. (**c**) Directivity pattern determined theoretically and from simulation; 3D scattering distribution intensity of far-field directivity pattern at different resonance frequencies with respect to increasing the chemical potential from 0 to 0.8 eV. (**d**) 2D projection of scattering intensity distribution of the far-field radiation lobe of the graphene gold nanostrip antenna at 10.3 µm. © 2018 Laser Physics Letters, IOP Publishing Ltd. Reprinted with permission from [139].

The geometric model is depicted in Figure 12a, where G1 and G2 are gold nanostrips with the length and width of 3300 and 300 nm, respectively, while ‘d’ is the gap between the two strips. Absorption, scattering and extinction cross-sections, as presented in Figure 12b, have a resonance peak at λ = 9.6 µm. It can be clearly observed that the absorption cross section is much lower than the scattering cross-section, resulting in greater scattering efficiency. When the chemical potential is changed from 0 to 0.8 eV, the resonance point shifts leftward in wavelength from 11 to 9.6 µm with decreases in the resonance peak strength. The sensibility of the refractive index and the switching of beam direction make this plasmonic antenna applicable in optoelectronics for the exploitation of light in nanoscale, as shown in Figure 12d. Controlling and tuning the scattering response of optical antennas by placing single- and multiple-layer graphene on top of an optical gold antenna result in a tunable wavelength in the visible to near-infrared region. Meanwhile, by adding the graphene sheet to the dipole antenna on the top, the resonance wavelength of the antenna shifts from 15 to 40 nm, as depicted in Figure 12c. The shift in the wavelength is directly proportional to the number of graphene layers placed on the antenna. The proposed method of resonance determined through the scattering characteristics of the graphene-based antenna could be exploited in biological and gas sensing applications [140]. The authors of [141] simulated the performance of a graphene antenna and employed the tunability by applying a gate voltage across the graphene sheet, which led to a reduction of the antenna size with a wide range of tunability over the infrared frequency range.

**Figure 13:**
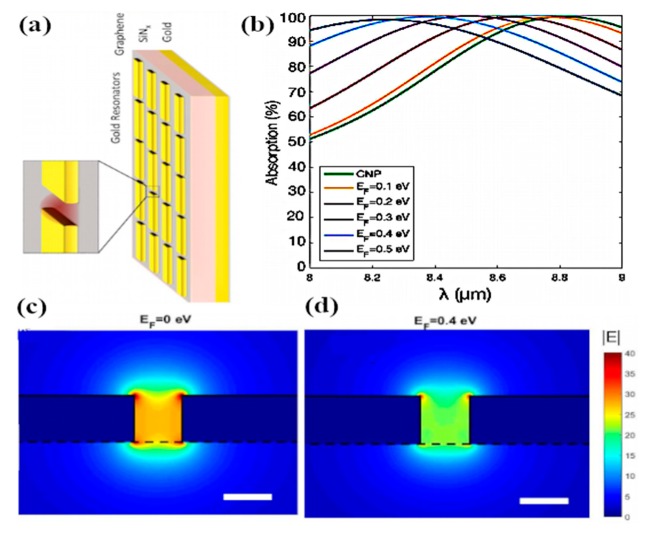
(**a**) Geometry of graphene–gold resonators, with graphene sandwiched between a silicon nitride substrate and gold nanoresonators; (**b**) Tunability of absorption at various Fermi energy levels; (**c**) EM field confinement at *E_F_ =* 0 eV at the gap between the adjacent gold resonators; (**d**) EM field concentration at *E_F_ =* 0.4 eV showing the detuning of resonance at different wavelengths. Copyright © 2017 American Chemical Society. Reprinted with permission from [143].

Naresh and colleagues investigated and developed a bowtie array antenna on the top of graphene sheets with the active swapping of a plasmon resonance at the optical region using a large graphene sheet [142]. The interaction of the graphene sheet with optical fields and a substantial effect at mid-infrared frequency occurs when the external electrostatic field is applied to change the Fermi level of the graphene sheet, thereby providing tunability of the plasmon resonance through electrical control. Moreover, Fourier-transform infrared spectroscopy (FTIR) is used for the characterization of this tunable antenna, as better resonance is achieved by reconfiguring the wavelength at the infrared region by increasing the gate voltage. In a subsequent work by Gonzalez et al. the launching and controlling of graphene plasmon propagation is demonstrated through the optical nanoantennas and tunable graphene sheet [143], as shown in Figure 13a. 

The optical antennas are tailored to launch and control the graphene plasmon which arises in the infrared region, and the refraction of these plasmons is analyzed when they pass through prism-shaped 2D bilayer graphene. This unique effect can be utilized for the manipulation of light in nanoscale optoelectronics and plasmonic circuits. The in-situ control of polarization and the phase of propagating far-field light by employing metasurfaces is demonstrated in [144]. Gold resonators with gate tunable graphene layers along with metasurfaces show 237° modulation of phase range at a resonance wavelength of 8.5 µm, which offers a tremendously reconfigurable reflected phase pattern at different wavelengths (Figure 13b). Applying voltage to graphene sheets provides a broad range of phase tunability from 0° to 206° with the shift in wavelength with a maximum resonance peak at 8.70 µm, as the varying voltage changes the Fermi energy of the graphene sheet, resulting in the EM confinement at the sharp corners of gold resonators (Figure 13c,d). Incorporation of antenna array with metasurfaces achieved 23% beam steering efficiency for the reflected light with the phase modulation range of 30°, thus approving the design of a graphene–gold antenna array integrated with metasurfaces for tuning the beam pattern in either direction at mid-infrared wavelengths. Furthermore, a 1% efficiency was achieved with the modulation phase up to 30°.

Yasir and coworkers experimentally demonstrated the tuning of frequency using multi-layer graphene in a nanoantenna [145]. The geometry of the antenna consisted of a rectangular radiating antenna along with a stub. The graphene layers were placed between the radiating antenna and the input of the stub. The authors confirmed that in the absence of the external gate voltage applied, graphene sheets behaved as an open circuit, restricting the passing of currents to the stub, and thus there was no effect of the stub on the performance of the antenna. However, when the voltage was applied and linearly increased, frequency reconfigurability was achieved over a wide range. In another work, frequency reconfigurability is demonstrated experimentally by tuning the reflection parameter through gate voltage [146]. Graphene sheets are embedded in center slot of the metallic antenna with coplanar formation. Using graphene in the center slot along with metalized absorber surface at the backside of antenna, wide bandwidth and better efficiency is achieved. For experimental demonstration, an antenna is fabricated on a 4-inch silicon wafer with silica grown by thermal oxidation up to 300 nm [147]. A chemical vapor deposition (CVD) process is used for growing graphene layers on the copper material, and is then transferred on to a SiO_2_ layer [148]. Raman spectra measurements are performed for the characterization of graphene, which validate that the CVD grown graphene is of good quality with few defects, while the measured reflection coefficient is in better agreement with simulation, having a small shift in the frequency. Ramin and coworkers showed the tunability of a parallel plate graphene photoconductive antenna (GPCA) by inserting a thin buffer layer in the gap between two graphene strips [149]. The strong coupling of graphene strips achieved better tunability at infrared wavelengths. Gradually increasing the electrostatic gate bias (i.e., changing the Fermi level) provides tunable GPCA, with an enhancement in the overall performance [150]. The concept of a graphene stack as a backing cavity is presented by Li et al., where the resonance of a graphene stack antenna with an incorporated Al_2_O_3_ layer between the graphene sheets depends on the graphene backing cavity [151]. Dual-band reconfigurability in a 1 THz wide band is realized by applying an electrostatic bias to the graphene backing cavity. In a subsequent study by Sasmita et al., bilayer graphene is utilized to attain the dual-band reconfigurability of a nanoantenna for terahertz sensing applications [152]. The field effect is used to control the conductivity of upper and lower graphene sheets dynamically, thus achieving dual-band tunability at terahertz wavelengths.

The performance of the graphene bowtie antenna are enhanced through the optimization of the substrate material and gradual tuning of the gate voltage in [153]. The tunability over the frequency region is accomplished by changing the chemical potential, while some characteristic parameters like gain and bandwidth are enhanced by optimizing the thickness of the substrate. In a subsequent work, the authors of [154] investigated graphene bowtie antennas for the enhancement of near-optical fields using a boundary element technique. The coupling of graphene plasmons excited in the gap region of the bowtie antenna produces high field intensity. Tunability over a wide range of wavelengths is shown by varying the Fermi level of the graphene sheets while keeping the dimensions of antenna fixed. By increasing the tip angle and radius of curvature of the graphene bowtie antenna, an enhancement occurs in the field intensity between the gaps of the bowtie. However, a bowtie antenna with rounded cornered triangles outperforms a simple bowtie antenna having sharp tips has better operational capabilities. It is found that by increasing the chemical potential of the rounded cornered bowtie antenna to 0.4 eV, a 220-fold enhancement of the field intensity is produced in the center gap of graphene bowtie. Penghong and coworkers replace the traditional metallic plasmons with graphene plasmons by exploiting the high tunability of ring-shaped optical graphene antennas [155]. Edge plasmons are produced in the perfect graphene ring-shaped resonators, while multipolar plasmon modes with anti-symmetric mode split are observed because of the breaking of symmetry in the nonconcentric graphene rings. Additionally, symmetric plasmon modes arising from the graphene rings have strong EM field concentration with an enhancement factor of 103 at terahertz frequencies, which is almost 20 times greater than the same size gold ring resonators. Xiaohe and colleagues demonstrated the concept of the graphene–metal hybrid antenna with circular beam tunability by using a loop-shaped metal antenna [156]. The metal loop antenna provides high effective radiation efficiency, and control of the dynamic surface conductivity by varying the gate voltage offers beam reconfigurability. Thus, combining the effect of both produces highly horizontal polarized 360° directional beams. Changing the position of DC gate bias pads of the rounded graphene sheet controls the resonance shift, which leads to a magnificent alteration of the beam pattern.

### 4.1. Tunable Absorption Handling in Graphene–Metal Antennas

Agreeing to the reciprocity statement, graphene-based tunable antennas are capable of transmitting and receiving electromagnetic waves from terahertz to optical wavelengths. Important antenna characteristic parameters (i.e., gain, impedance, radiation beams) are the key elements for the antenna to work as a receiver. However, apart from these parameters, optical absorption performs a significant function, aimed at the conversion of free space propagating waves into surface waves with high responsivity and better enhancement in the near field [157]. The variation and improvement of absorption in a graphene-based antenna will allow the development of new methods to control antenna performance with more insight into the design structures.

Pure single-layer graphene has an optical absorption ability of 2.3%, which is quite unsatisfactory to apprehend intense interactions of light and matter. Different methods have been employed to enhance the optical absorption of graphene antennas and likewise couplings effects, photonic modes and graphene plasmons. For example, varying graphene chemical potential through the electrostatic bias of a tooth-shaped log-periodic graphene antenna achieved tunable absorption at infrared wavelengths as well as high reconfigurable intensity [158]. Electrostatic biasing of graphene in nanodisk arrays and optimizing the separation space between neighbor disks produced a 30% enhancement in graphene absorption when compared to the pure graphene absorption [159]. Active tuning of the absorption in graphene disk arrays is controlled by varying the chemical potential of graphene sheets. In another work, the authors of [160] experimentally proved the strength and energy of plasmons by tuning the optical absorption in a disk array having a single graphene sheet. The effect of critical coupling is theoretically investigated to attain perfect absorption in graphene sheets by using photonic bandgap and substrate grating, and thereby unity absorption in graphene is achieved [161]. Besides, the integration of graphene with metallic nanoantenna structures offers more openings to design and engineer the absorption properties of graphene. Qin and coworkers demonstrated the enhancement of optical absorption in graphene integrated nanostructures using the concept of trapping energy in cross-shaped structures [162]. The interaction of a metallic split cross antenna with a graphene sheet provides 30% absorption and tuning of the resonance wavelength in a broad range from 780 to 1760 nm when different dimensions of cross split structures are used. This significant improvement of absorption and efficiency in graphene spilt cross antenna can be used in applications like graphene solar cells and photodetectors.

**Figure 14:**
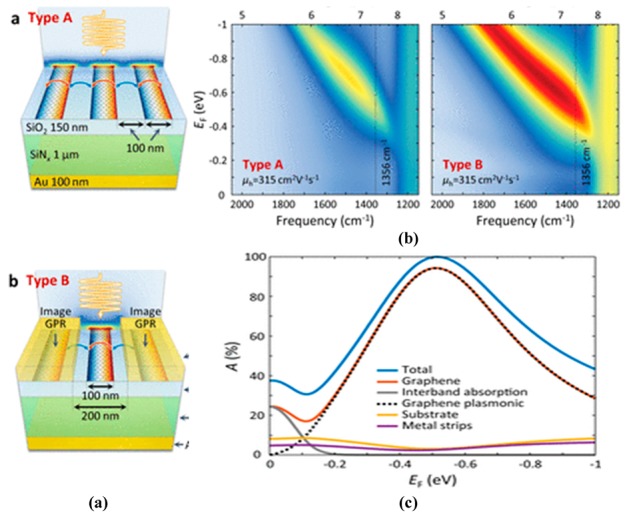
(**a**) Different geometries of graphene plasmonic resonators; (**b**) Mapping of absorption with respect to frequency and Fermi level; (**c**) Absorption tuning considering the different conditions as a function of Fermi energy, as better absorption is achieved at *E_F_* = -0.514 cm^2^V^−1^s^−1^. © 2018 ACS. Reprinted with Permission from [166].

Yu et al. demonstrated a model for complete absorption using a graphene layer. Complete absorption takes place due to destructive interference between the reflection of a radiating surface and a back reflector [163]. However, destructive interference of the reflections and transmission produces almost zero absorption. Employing a back metal reflector in a graphene antenna results in the tuning of the resonance mode from zero to complete absorption. Changing the chemical potential of graphene also produces a resonance shift because of the destructive interference. In addition, a stacked graphene substrate is employed to overcome the problem of resonance shift and acts as plasmonic bandgap structure, thus actively tuning the phase of reflection by tuning the Fermi level of the graphene stacked substrate. Mackenzie et al. worked on a graphene broadband tunable antenna based on a metamaterial absorber, and achieved approximately 90% absorption in the region from 0.65 to 1.3 THz [164]. Furthermore, the transmission line model is adopted to study the impedance behavior of a metamaterial absorber and tuning the absorption bandwidth by varying the chemical potential of graphene layers. Optical absorption in a single-layer graphene integrated optical antenna configuration through efficient electrostatic tuning is reported in [165]. Electrical tunability is accomplished by incorporating an ionic gel which acts as a gate dielectric and a vertical micro-cavity known as a Salisbury screen, which is modeled by two dielectric transparent spacers and arrays of inverted gold resonators. The coupling of plasmons between the gold resonators and the graphene layer produces enhanced optical plasmons. Following this, these plasmon excitations are electrically tuned by decreasing the chemical potential, thus allowing high absorption intensity to be attained. Seyoon and coworkers came up with a solution to enhance the low coupling of graphene plasmonic resonators and poor carrier mobilities which lead to imperfections in graphene [166]. By tuning the optical absorption in graphene resonators, the authors experimentally proved 96.9% absorption at 1389 cm^−1^ with a modulated reflection efficiency of 95.9%, as depicted in Figure 14c. Furthermore, authors demonstrated the tuning of active absorption in graphene electronically, covering 10% less surface area with the incorporation of a low-permittivity dielectric substrate and gold-based metallic nanoantennas to boost the deeply coupled radiation of graphene resonators. Figure 14a illustrates the different geometries of graphene resonators, where “type a” has only graphene resonator strips and “type b” has graphene integrated with gold resonators.

### 4.2. Modulation of Optical Features by Tuning Graphene Conductivity

Electrically changing the chemical potential of graphene with the gate bias progressively transforms its dielectric constant, hence playing a significant role in the continual modulation of antenna parameters [140,167,168]. Renwen and coworkers proved an outstanding modulation of optical light transmission greater than 90% in a graphene-based silver nanoantenna [169]. The tuning of plasmons through on- and off-resonance produce strong variations in the absorption and reflection of the incident light. Furthermore, a spectacular >600% enhancement of reflection is attained by increasing the thickness of the silver in the graphene loaded antenna up to 5 nm. Yu et al. accomplish a better modulation of infrared light for both amplitude and phase through active resonance shifting by electrically tuning the graphene conductivity [170]. A total of 240° phase modulation with a modulation intensity of 100 is realized systematically through light scattering from antenna arrays. FDTD simulation is adopted for the optical response and the graphene layer is modeled as an isotropic material. However, a random phase approximation model is implemented for the calculation of graphene’s optical conductivity. The intensity and modulation depth vary in accordance with the tuning of the graphene conductivity. Furthermore, the backplane resonator concept is employed to achieve sharp resonance, thereby producing better coupling between the rod antenna and the image rod on the back of the antenna. The interaction of graphene with light can be enhanced by combining graphene plasmons with metallic plasmons and the active control of optical responses. The authors in [171] worked on the integration of metal–insulator–metal (MIM) waveguides in a graphene antenna in order to actively tune the conductivity of graphene. Electrically changing the conductivity enables tunability for a wider range up to 1100 nm, with a 20% enhancement of the resonance frequency in the mid-infrared wavelengths in a coupled graphene antenna along with integrated MIM waveguides. To date, graphene has been used as an active medium to modulate the optical characteristics of plasmonic devices, but the applications which need tuning and controllable magnetic responses have yet to be explored. In a recent work, Ningbo and coworkers explored the magnetic resonance in graphene–gold nanoantennas [172]. Optimizing the geometry of graphene–gold diabolo antennas is best fit for tuning the magnetic response in the infrared region with wavelengths from 32.3 to 19.8 µm. The magnetic responses are one order of magnitude higher than simple metallic diabolo antennas, and an enhancement of 12% in the magnetic field with an absorption modulation of 1460% is achieved. In another work, Guozhen and colleagues demonstrated a graphene modulator with 100% modulation depth and efficiently modulated the intensity of terahertz light from an incident quantum cascaded laser [173].

**Figure 15:**
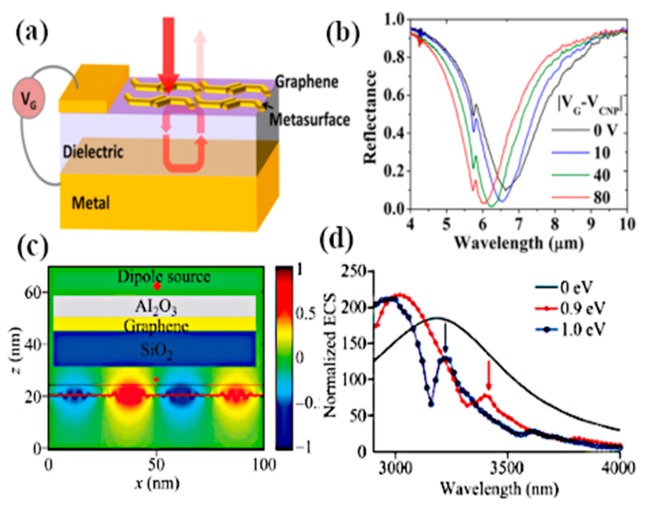
(**a**) Illustration of graphene-antenna-integrated metasurfaces. Antenna arrays are placed on the top of the graphene sheets, and dielectric acts as an optical cavity for incident radiation; (**b**) Tuning of resonance wavelength with increasing external gate voltage; (**c**) Distribution of near field as a thin metal oxide layer of Al_2_O_3_ is inserted between graphene and a dipole source; (**d**) Normalized extinction cross section of the on/off mode coupling at different chemical potentials. © 2014 ACS, © 2016 Springer Nature. Taken and reprinted with permission from [174,184].

Dong and coworkers demonstrated the enhancement of graphene light interaction using coupled resonance with higher modulation by exploiting as cross-shaped slot metamaterial absorber [174]. Blueshift occurs in the resonance frequency with increasing Fermi level, and there is a wide range of tunability in the terahertz region as a result of having highly modulated optical characteristics (Figure 15b). Electrically driven vanadium dioxide (VO_2_) metal–insulator phase transition is studied experimentally for the broadband modulation of light in the terahertz region [175]. The plasmonic modulator is composed of bowtie array antennas with active layers of vanadium dioxide, which provide tunability by applying an external gate voltage to electrically change the phase transitions. First, the modulation depth is enhanced by placing metal wires on the VO_2_ surface, and then modulation of terahertz light is further improved by adding a bowtie antenna between the wire and the VO_2_ layers. Yu et al. demonstrated the realization of metasurfaces for tuning optical ranges and modulation depths [176]. The metasurface structure was composed of metallic antennas on the top of a graphene sheet, which was incorporated in a subwavelength optical cavity to design the perfect tunable absorber illustrated in Figure 15a. The critical coupling condition was exploited by changing the voltage across the gate, thus acquiring a high modulation depth up to 100% over a wide bandwidth of 5–7 µm wavelength. Fano antennas based on split ring resonators [177] nanorod gold antennas [139] and optical Yagi-Uda antennas [178] have been theoretically studied and demonstrated for the modulation of antenna characteristics (i.e., directivity, radiation efficiency and beam pattern) by manipulating the chemical potential of graphene upon applying different gate biases. Besides, in some RF and terahertz patch antennas, graphene material is adopted as the photoconductive source the providing dynamic control of modulation with stimulating capabilities [139,179].

### 4.3. Effective Utilization of Graphene Plasmons

Surface plasmon polaritons (SPPs) propagation maintained by single-layer graphene can be vigorously tuned by changing the Fermi level by electrical doping, hence enabling the design of resonant spectral location and their interface with the exciting metallic plasmons. Earlier reports exposed the good coupling behavior of graphene plasmons with metal plasmons arising from the thin metal layer, with an enhanced performance [180]. The authors demonstrated that plasmons arise from the monolayer graphene sheet and plasmons from a fragile metal sheet having a thickness of tens of nanometers have comparable plasmonic properties. A new type of resonance behavior is observed in graphene nanoantennas when the layers of the graphene sheet are interconnected with gold metal [181]. Tunable plasmonic waveguide channels can be easily inserted in these antennas with electrostatic bias pads because they offer new type of plasmonic modes. In addition, when the graphene sheet was excluded, it proved that the inductive behavior of graphene is a crucial component to sustain plasmonic resonance. Thus, the spectral resonance width declines contrariwise with the charge carrier mobility. However, when graphene is placed in contact with metal, the plasmons become naturally radiative and have a tunability in transmission of 100% at the resonant wavelength by changing the carrier density in graphene. Zhao et al. proposed optical plasmon-induced transparency by designing a novel metamaterial structure containing single-layer graphene [182]. Destructive interference between dipole and monopole antennas produced peak transparency in the transmission spectrum. Tuning the Fermi energy level of graphene strongly controlled the spectral width and line shape transparency. Active control of propagating SPPs could lead to the actualization of innovative optoelectronic devices. Yanjun and coworkers developed a graphene device to launch unidirectional SPPs and electrically control the propagation of SPPs [183]. The structural geometry of a graphene-based field-effect transistor device is comprised of a reflective antenna pair (RAP) design, acting as the source and drain electrodes. Upon applying voltage to the electrodes, the optical resonance is tuned and the device behaves as a unidirectional SPP launcher. Xingang et al. proposed a metal–insulator graphene nanoantenna to tune and control the near-field distribution characteristics of a dipole antenna [184]. Tuning the chemical potential of a graphene sheet separated from the metallic contact by an insulator modifies the dispersion relation with a strong coupling on the surface of the insulator between the graphene plasmons and metal plasmons. Both the in-phase and out-of-phase coupling produce a shift in the resonant wavelength, with enhanced far-field and near-field responses. Furthermore, the quantum tunneling effect of graphene with metal is reduced by inserting a thin metal oxide layer between graphene layers and the metal dipole antenna (Figure 15c). The results show a better coupling of graphene plasmons, which results in the resonance splitting of a metal dipole antenna under the proper chemical doping of graphene. Electrically applying voltage to tune the Fermi energy of graphene can efficiently switch (on/off) the mode coupling, as presented in Figure 15d. Lastly, all the graphene antennas discussed in Section 4 is summarized in Table 2.

## 5. Graphene Leaky-Wave and Reflectarray Antennas for Frequency Tuning and Radiation Beam Scanning

Fascinating beam steering properties of leaky-wave antennas (LWAs) have been an exceptionally active area of antenna investigation in previous decades [186,187,188,189,190,191]. The actual mechanism on which leaky-wave antennas are based is the radiation leaking from the propagating electromagnetic wave in the waveguide medium towards free-space, where the phase velocity of the leaking radiation is greater than the speed of light. Even though most leaky-wave antenna designs have been intended to work at miniaturized scale and with millimeter waves [192], their hidden science is an essential key to clarify colorful physical ideas like surprising transmission [193], Cherenkov radiation [194] or electromagnetically tempted transparency [192]. Graphene is especially appropriate for controlling the propagation and radiation of leaky-wave antenna at THz and infrared wavelengths, permitting the actualization of tunable designs and—even more significantly—the accomplishment of extraordinary non-reciprocal magnetic responses. The first concept of influential leaky-wave antennas was introduced in the 1950s [195], and is based on the modulation of reactance surfaces. It obtained noteworthy consideration at microwave frequencies [196,197,198]. Floquet harmonics are produced due to periodic modulation, which permits the expression of the EM field as an infinite sum of harmonics on the top of the surface, where some of them may exist in a light cone and become leaky waves, causing the waveguide design to function as an antenna [199]. The fundamental parameters controlling the performance of leaky-wave antennas are the modulation period *p,* modulation amplitude *M*, and the average reactance *X_s_*. The reactance should be inductive in order to sustain highly confined TM waves that powerfully interact with the modulated surface. The exact values of modulation amplitude, radiation rate, and angle regulate the field confinement and wave propagation constant. Equation (13) represents the expression of modulated surface reactance:

(22) $$X_{s}^{\prime }=X_{s}\left[1+Msin(2\pi y/p\right]$$

**Figure 16:**
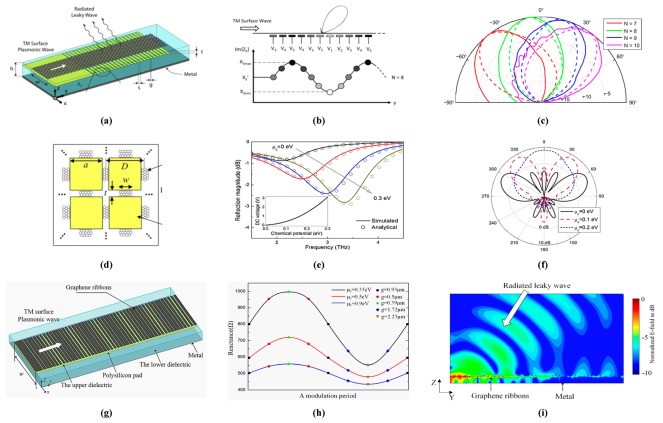
(**a**) Schematic of a graphene leaky-wave antenna (LWA), with a graphene sheet placed on top of gating pads. (**b**) Representation of the graphene reactance with respect to DC voltage at the gate pads. (**c**) Radiation beams of the LWA; as the parameters are changed by applying gate voltage, the beam steers in either direction, keeping the distance between gate pads constant (i.e., g = 0.2 µm). (**d**) Geometry of a graphene-integrated leaky-wave antenna with metasurfaces. (**e**) Tuning of reflection magnitude through simulation and analytical means; when the chemical potential is increased from 0 to 0.3 eV, the resonance peak shifts to higher frequencies with increasing reflection magnitude. (**f**) 2D radiation pattern of the integrated high-impedance surface (HIS) antenna with beam scanning ability as the chemical potential is raised. (**g**) Geometric representation of sinusoidally modulated LWA; graphene nanoribbons with unequal strip width are placed on the polysilicon gate. (**h**) Modulated reactance of LWA by altering the parameters of the graphene and the space between graphene sheets. (**i**) Electric field pattern of the radiated beam from the LWA. © 2013 OSA, © 2015 IEEE © 2018 APS. Reprinted with permission from [199,200,203].

Graphene is the ideal stage to actualize this idea at THz and infrared frequencies. It provides an inductive behavior, given as $Z_{s}=1/\sigma =R_{s}+jX_{s}$, and by employing the graphene field-effect, the surface resistance and reactance of the graphene sheet can be manipulated [25]. Moreover, in controlling the reactances of the graphene leaky-wave antenna by applying electric voltage, excellent features of the antenna (i.e., beam steering and radiation pattern reconfigurability) can be efficiently tuned. In 2014, Esquius and colleagues proposed the first leaky-wave antenna [200] based on a graphene strip, depicted in Figure 16a. The design they investigated is comprised of a graphene sheet placed on top of the substrate. The substrate itself is back-metallized and has polysilicon DC gate pads underneath the graphene sheet. By electrically applying regulating voltages to all polysilicon gating pads, arbitrary reactance modulation can be actualized on the graphene sheet, thereby permitting leaky-wave spread and propagation. Notably, altering the characteristic parameter of modulation progressively controls the radiated beam spilling rate and pointing angle. The voltage applied to DC pads eventually depends on the capacitance of the substrate and the reactance of the graphene sheet. Figure 16c represents the exceptional abilities of the leaky-wave antenna. By varying the gating voltage at the DC pads, the radiation pattern changes direction from backwards to forwards. The lossy nature of graphene plasmons reveals that it is challenging to obtain better efficiency and highly directive radiated beams concurrently, and so one might trade up with one feature. Using a high value of *M* for the effective aperture will bring improved efficiency, and vice versa. However, this configuration simultaneously has 11% radiation efficiency and beam reconfigurability. In a subsequent work by the same authors [201], a sinusoidal-shaped leaky-wave antenna is investigated. The antenna is excited by using the periodic modulation of graphene strips without gating pads, thereby exploiting the huge affectability of plasmon propagation relative to the width of the graphene strip in order to produce spatial leaky waves. For simplicity of computation, the modeling of this graphene leak-wave antenna design would exploit the effective modulation of the reactance, plotting the plasmon dispersion (which is dependent on strip width) to the actual fluctuations in graphene conductivity. Such investigation can be proficiently performed by utilizing the scientific scaling law of SPPs versus strip width, and the design this sort of antenna is realized in [201]. Although the implementation of this geometry is simple to fabricate because there are no DC pads beneath the graphene sheet, the less control of the modulation amplitude results due to the static frequency of operation.

Wang and co-authors demonstrated a graphene-based 2D leaky-wave antenna with simultaneous beam steering and frequency reconfigurability features in the terahertz frequency region [202]. Graphene sheets were employed to tune the high-impedance surface (HIS) which acted as the ground of the proposed antenna illustrated in Figure 16d. Altering the gate voltage produced changes in the conductivity of the graphene sheet, and tuning the chemical potential significantly changed the HIS reflection phase, thereby controlling the resonance frequency peak movement at the terahertz region (Figure 16e). However, the beam radiation pattern was effectively tuned in different directions, keeping the resonant frequency constant by increasing the chemical potential, as depicted in Figure 16f. In another work by Zhan et al. the idea of a dielectric grating geometry is implemented to accomplish tunability of a leaky-wave antenna in the terahertz band [203]. Two different methods are adopted to eliminate the mismatch between SPP and free space wave numbers, and to effectively radiate SPPs into free space. In the first scenario, a graphene sheet is considered as being suspended and supported by a silicon nitride layer at the grating structure, while in the second scenario graphene is placed at the interface of the substrate and air. Applying a bias voltage at the gate significantly controls the graphene SPPs, while the dielectric grating is responsible for exciting leaky modes, thus effectively tuning the radiation pattern of the beams in the terahertz frequency range.

The novel concept of mantle cloaking is adopted for the beam steering of a leaky-wave antenna in [204]. The authors wrap the antennas in tuned graphene sheets to reduce the mutual coupling between the antenna and to suppress the EM interaction of the radiating antennas. Tuning the chemical potential of the wrapped graphene sheets reduces the mutual coupling and thereby provides beam reconfigurability over a wide range. Yan and coworkers present the sinusoidal modulation of a leaky-wave antenna by employing only one bias voltage to tune the beam steering property of an antenna when voltage is applied at the gate pad [205]. The geometry of the proposed LWA is depicted in Figure 16g. The design structure is comprised of unevenly spaced graphene nanoribbons placed onto a polysilicon gate pad. The unevenly spaced GNRs support leaky-wave modes by sinusoidally modulated surface reactance as shown in Figure 16h. Increasing the chemical potential effectively controls the leakage rate with the change in the direction of the radiated beam.

Different ways to actualize leaky-wave antennas in the optical region require borrowing basic principles from microwave theory, such as circularly polarized antennas and transmission lines. In particular, the authors of [206] considered the suitability of actualizing such strategies at THz frequencies utilizing graphene plasmons. Even though the fundamental outcomes are to some degree promising, the fabrication of LWAs is difficult and requires high-quality graphene, which may postpone its practical utilization. In a different context, versatile vibrations based on flexural (mechanical) waves have likewise been proposed as an strategy to excite LWAs by periodically modulating reactance, completely manipulating the multifaceted behavior of graphene [207]. In this approach, the propagation of flexural waves is dependent on the graphene surface, which can be precisely modeled using static grating. Altering the frequency of mechanical waves with biharmonic sources will progressively control the radiated beam reconfigurability. Additionally, the proposed antenna can provide any modulation reactance profile, on account of the adaptability of mechanical waves. Thus, it can execute the desired beam pattern in multiple directions. However, the fabrication of plasmonic devices and their integration with mechanical components is challenging in realistic use. Another way to exploit graphene leaky-wave antennas is by utilizing graphene as an agitation element, as already discussed in resonant antennas [208]. The inspiration is to manipulate the benefit of various innovative technologies to expand and provide high performance or decrease the dependence on high-quality graphene.For instance, in [165] and [185], graphene is constantly cast off to give quasi-real-time electronic tunability that would be practically difficult to accomplish with traditional materials. The same situation occurs in leaky-wave antennas incorporated with high-impedance structures to provide high tunable performance and beam steering due to the satisfactory utilization of graphene [209,210]. Fuscaldo and coworkers implemented an approach where graphene is incorporated between substrate-superstrate LWA to permit static frequency beamforming [211]. Here, the graphene sheet is streamlined with the end goal that it fundamentally influences the propagation constant of leaky modes. By cautiously optimizing the geometry of graphene substrate-superstrate LWA, the design attains high reconfigurability, which limits the spillage rate and enhances the directivity when producing tunable radiated beams. In changing the chemical potential from 1 to 0.3 eV through the gate voltage, the antenna exhibits reconfiguration abilities with 45° beam steering. In the most recent subsequent work by the same authors, they stressed that the critical parameter is the relaxation time of the graphene sheet, which directs the radiation functioning of LWAs (either plasmonic or non-plasmonic). For this reason, they proposed and experimentally evaluated a Fabry–Perot cavity leaky-wave antenna [212]. First, the modeling of the antenna along with an open-ended waveguide THz source was validated through FDTD simulations, and then a cylindrical geometry was adopted to exhibit an omnidirectional radiation pattern with broadside beam steering of 45°. Second, graphene samples were characterized by THz measurements and the relaxation time was measured by adding the transmittance measurement. In a related study [213], transverse mode is adopted for the Fabry–Perot cavity (FPC) antenna. The radiation operation of the FPC is dependent on the excitation of cylindrical leaky waves. This approach accomplished much higher radiation efficiencies than simple graphene-based radiators which excite SPPs in leaky regime. Furthermore, in [214] a graphene metasurface is incorporated in a multi-layered substrate structure to achieve a high-performance FPC leaky-wave antenna. Notably, the metasurface in the FPC antenna reduces the ohmic loss and systematically enhances the radiation efficiency while imparting reconfigurable beam features [194].

In another setting, an ongoing and extremely encouraging trend in both the EM and antenna arenas is the interest in non-reciprocal response without magneto-optic properties which require bulky magnets to function, thereby restricting the miniaturization of plasmonic devices and their applicability in other electronics [42]. This is particularly significant on account of THz graphene plasmonics, where the subsequent segments are altogether smaller than the corresponding magnets required in silicon applications. As a novel standard, it has recently been adopted that the time-reversal symmetry be broken by modulating the features of the antenna in space and time. Correas and coworkers [214,215] adopted such ideas, which were converted into graphene plasmonics in the terahertz region by sufficiently manipulating the magnificent reconfigurability of the material through its field impact. In particular, this methodology empowers the advancement of THz antenna parameters with various transmitting and receiving properties [216]. More interestingly, transmitted/received SSPs oscillate at various frequencies under time reversal. The blend of these two marvels gives rise to exceptional non-reciprocal response in the specific circumstance of LWAs, as some of the same methods are practiced for the development of LWAs in the microwave domain [43,216]. Furthermore, these non-reciprocal responses of terahertz LWAs are based on the spatiotemporal modulation of the graphene conductivity [36]. Non-reciprocal responses are observed when different bias voltage signals are applied to gate pads, thus each one has a different oscillating frequency, creating various conductivity behaviors across the graphene sheet, providing a spatiotemporal modulation of conductivity. This phenomenon generates and provides an active medium for propagating plasmons to break the time-reversal property, enabling the antenna to operate in single mode (either transmit or receive mode). The interest in the non-reciprocal devices by the optoelectronics community will increase in the coming years due to the space-time modulation of graphene conductivity which will bring a revolution in plasmonic systems and antennas for various applications in the optical regime.

### 5.1. Graphene Reflectarrays for Vortex Beam Generation and Beam Scanning

Reflectarrays have been a hot topic among multiple domains (e.g., RF, microwave communications and antenna design communities) [217,218]. Moreover, they have empowered numerous satellite correspondence and terrestrial microwave communications with exceptional gain capacity in the most recent decades [219,220]. Reflectarrays consist of an exciting feed antenna and a broad array of reflective unit cells. When the reflective array is excited by the feed antenna, each element in the reflectarray produces phase-shift due to the reflection of the wave on the array surface [219]. The collective impact of all array elements permits the control of the reflected radiation phase fronts, thereby producing high directive far-field radiation beams in almost every direction. It is significant to note that reflectarray and transmitarray operations are based on “generalized Snell laws”, which has provided the basis for the stimulation of plentiful research in the field of optics in previous years [221,222,223,224]. The unusual mechanism of reflectarrays consolidates the benefits of phased array antennas and parabolic reflectors, producing sharp and reconfigurable beams from low-profile planar geometries with a simple feeding network relative to traditional antenna arrays. Various advancements have been proposed to progressively control the phase of the single element at a miniaturized scale in the microwave and millimeter-wave frequencies, for example, MEMS [225,226,227] and semiconductor diodes [228,229]. However, such large structures cannot be employed in the terahertz region due to size issues and high coupling loss. Graphene is a hopeful possibility to fill this void because its high inductance and the substantial electric field impact result in the possibility of scaling down and the expected tunability.

The groundbreaking investigation on the graphene-based reflectarray has been firstly presented in the year 2013, by Carrasco and Perruisseau-Carrier [230]. The reflectarray proposed by the authors is comprised of graphene patch unit cells moved onto a silicon dioxide substrate (SiO_2_). In a traditional reflectarray unit cell patches are designed using a metallic material and noble metals. The metallic patches resonate when their physical size is equivalent to a half wavelength of the wave in an actual medium, thereby leading to large geometric structures. This situation becomes diverse when graphene patches are considered, as the graphene patches resonate at much lower frequencies because of the slow propagating plasmonic modes of graphene when compared to their metallic counterparts. However, graphene plasmonic responses prompt a sharp decrease in the electrical size of the array elements, providing overall better performance. For instance, the reflectarray in [230] has graphene patches sizes equal to λ/16, thus permitting miniaturization.

**Figure 17:**
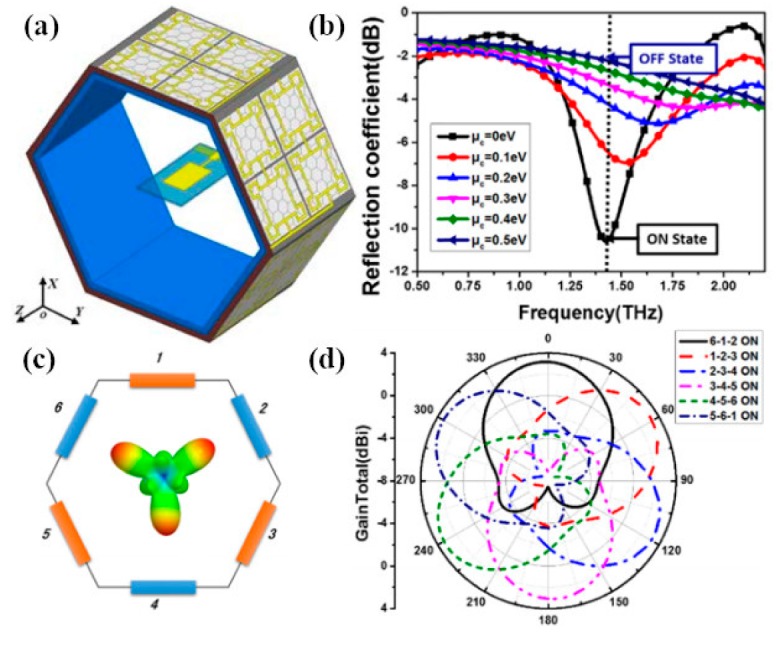
(**a**) Monopole THz antenna integrated with reflective frequency-selective surfaces; (**b**) Magnitude of the reflection coefficient by increasing the chemical potential and consecutively switching the frequency-selective surfaces (FSSs) on/off; (**c**) Schematics of the switch on/off scenario for FSSs; (**d**) 360° radiation beam scanning by alternatively switching the FSSs on/off. © 2018 OSA, Reprinted with permission from [235].

The total reflectarray is formed from 25,000 graphene elements and is fed through a terahertz horn antenna. Moreover, applying DC bias voltage at the polysilicon gate layer effectively tunes the graphene sheet reactance and accordingly the phases of the array elements. Importantly, the miniaturized size and tunability of beams concerning the phase response of graphene patches is the most significant advancement in reflectarray antennas [231]. However, more fascinatingly, the tunability and size of graphene reflectarrays is dependent on chemical potential; using low values of chemical potential produces more significant and dynamic phase variations. In another study, Carrasco demonstrated a reconfigurable terahertz graphene-based reflectarray for the implantation of the generalized law of reflection [232]. The conductivity of graphene was tuned through gate voltage control, the reflection phase and the radiation beam direction of the array. Using appropriate chemical potential values, the authors achieved a wide 300° phase tuning range and low coupling loss between array elements with a smaller separation space. In a recent study, Forouzmand and Mosallaei demonstrated the beam steering property of tunable reflectarray metasurfaces by integrating a thin indium tin oxide layer in a plasmonic unit cell array [233]. By electrically varying the bias voltage, the resonance properties and buildup of carrier density at the boundary of indium tin oxide result in 250° beam scanning at 218 THz. Wu et al. proposed the active tuning of a radiated THz antenna beam in 360° by employing graphene–gold frequency-selective surfaces (FSSs) around the antenna geometry [234], as depicted in Figure 17a. Altering the chemical potential of graphene and alternatively switching reflective selective surfaces around the omnidirectional monopole antenna resulted in the steering of the beam as presented in Figure 17c,d, by providing 360° beam scanning. When FSSs 3, 4 and 5 were on, the maximum resonance of the antenna was observed at 1.4 THz (Figure 17b). In addition, both the transmission and reflection coefficients of the frequency selective surface are reconfigurable because of the varying graphene chemical potential, which allows control of the radiation gain and beam direction.

The generation of vortex beams is the most common application of reflectarrays. Vortex radiation beams are described by orbital angular momentum (OAM), which allows the spectral efficiency to be increased by utilizing the originality of a vast number of phase states. Vortex beams have attract a great deal of interest in previous years on account of promising examinations, guaranteeing that OAM may give improved transmission characteristics compared to some other communication technologies [235,236]. Although OAM multiplexing is a subset of MIMO devices and consequently has less gain capacity than MIMO devices [237], vortex beams have stimulating utilization in the field of communication with improved encryption abilities, laser detection and optical energy trapping [238]. However, the formation of such beams is demanding. One typical method to generate vortex beams from reflectarrays is by manipulating the reflection from plane-waves. The plane-wave strikes over the whole surface area of the array, with every sector having equivalent reflection magnitude. Whereas, the order of the vortex beam can be determined from the addition of phase increments to the multiple of half-wavelength. Moreover, graphene patches are a superbly suitable platform for the generation of vortex beams, along with more considerable design adaptability.

**Figure 18:**
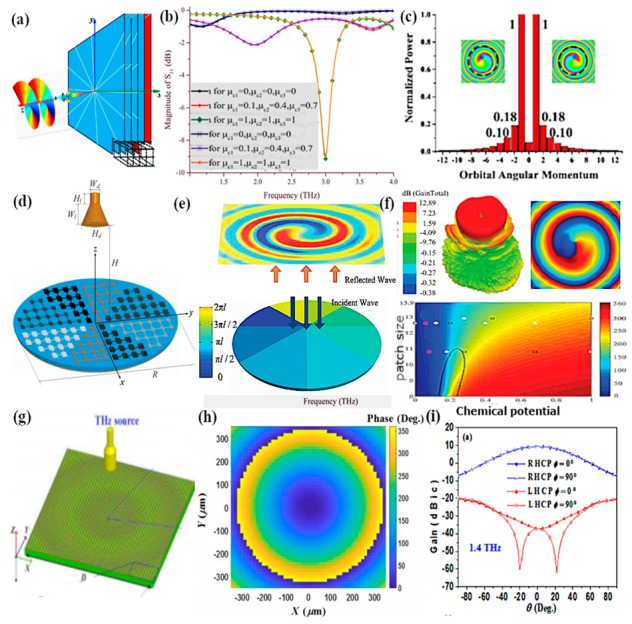
(**a**) Schematics of graphene reflectarray geometry for the generation of vortex beams; the whole reflectarray is divided into sectors for the different phase tuning. (**b**) Resonating frequency of the proposed graphene reflectarray, by proper tuning of the chemical potential. (**c**) Orbital angular momentum at different vortex modes. (**d**) Geometry of a reconfigurable graphene reflectarray antenna, where dark and bright sectors of the graphene patches correspond to different chemical potentials. (**e**) Reflective surface model for the generation of vortex waves; different sectors have varying phase when excited by a plane wave. (**f**) 3D radiation gain pattern showing a high achieved gain of 12.89 dBi and reflection coefficient with various patch sizes and chemical potential values. (**g**) Structure of circularly polarized graphene reflectarray with circularly polarized source. (**h**) Distribution of the reflected phase of the reflectarray employing a metasurface. (**i**) 2D radiation pattern of the circularly polarized graphene reflectarray at 1.4 THz; the blue line represents the right-handed circular polarization (RHCP) and red lines represent left-handed circular polarization (LHCP). © 2019 OSA, © 2018 AIP, © 2018 MDPI. Reprinted with permission from [240,241,243].

The recent investigation by Yanshi et al. developed a graphene-based reflectarray with metamaterial structure for the production of (OAM) vortex beams at terahertz frequency [239], as depicted in Figure 18a. The reflectarray design is comprised of multilayer graphene metasurface patches placed on a silicon dioxide substrate, and a thin oxide insulator layer is inserted between them. The ground plane is metallized by gold to function as a reflective layer. DC voltage is applied across the graphene layers and SiO_2_ to adjust and tune the chemical potential of graphene. Increasing the chemical potential of graphene layers results in an improved reflection magnitude of −2.5 dB with 360° phase reflection range, as illustrated in Figure 18b. Furthermore, by selecting the proper and accurate values of chemical potential, the proposed reflectarray produces OAM vertex waves having three different modes, l = ±1, ±2, ±3. In addition, the OAM beams of the reflectarray function in a wide frequency range of 1.8–2.8 THz with better tunability and generating OAM vortex waves with the highest spectral intensities as shown in Figure 18c. In the related context, Zhuang and coworkers presented the production of OAM vortex waves utilizing a reconfigurable graphene reflectarray [240]. A circular reflective sectored geometry was adopted for the generation of vortex waves at terahertz frequencies by using graphene patch arrays at different sectors in a circular manner (Figure 18d). The reflection coefficient of the circular reflectarray is tuned through varying chemical potential and changing graphene patch geometry. Figure 18e represents the circular reflective sectored model for the generation of vortex waves when the plane wave is incident on the circular reflective sectored surface. Thereby, all sectors produce reflections of the same magnitude, but the phase variation is increased by a multiple lπ of 2π over one circle. In addition, to obtain desired modes of the vortex beams, the geometries of the graphene patches should be fixed, while chemical potential must be varied by the bias voltage to achieve the mode reconfigurability of the reflectarray as illustrated in Figure 18f. Upon exciting the circular reflectarray with a horn antenna, the mode of vortex waves at 1.6 THz can be alternatively tuned to various modes (0, ±1, ±2). 

In a related study, Jian et al. demonstrated a new technique for the generation of vortex plasmons through cross-shaped metal–graphene nanoantennas [241]. To excite cross-shaped nanoantennas, the linear polarization incidence is exploited, and thus the creation of graphene near-field plasmons vortex are the result of the coupling of each cross-shaped patch in the array at the resonance frequency. However, by arranging the cross antenna in a circular array configuration, the vortex plasmons can be subsequently reconfigured by absolute field distribution and by the direction of linearly polarized incidence. Furthermore, the gap between graphene and metallic nanoantennas are kept small for coupling effects. The authors stressed that shortening the gap between graphene and cross-shaped antennas will result in higher coupling efficiency with an unremarkable effect on the field confinement at the center of the cross-nanoantenna array. However, in a related context, the shortening of the gap affects the phase difference of plasmons generated by the arms of the cross antenna. Due to the short interspacing distance, the antenna design requires optimization to produce vortex plasmon.

Li Deng and coworkers theoretically inspected a circularly polarized graphene-based reflectarray to achieve wide bandwidth and better gain in the terahertz region [242]. The investigated graphene-based reflectarray was excited by a terahertz circularly polarized source (Figure 18g). The concept of the Pancharatnam–Berry phase is realized to achieve a tuning phase range of 3600 in the bandwidth of 1.4–1.7 THz by rotating the geometries of graphene unit cells in the array to overcome the problem of graphene’s narrow-band resonance. According to the optical path reversibility principle, metasurfaces are utilized as a focusing surface when the reflectarray is incident by the circularly polarized source [243]. Figure 18h illustrates the 360° phase tunability of reflectarray unit cells. Furthermore, the proposed reflectarray with integrated metasurfaces achieved better gain and highly circularly polarized stable radiation beams with an axial ratio of 2.1 dB, as described in Figure 18i. Ben et al. demonstrated a switchable focus for incident spherical waves to excite a circular graphene reflectarray [244]. Electrically controlling switchable focus and changing the chemical potential of graphene provides a peak gain of 22 dBi with 65% beam radiation efficiency at 1.5 THz. In another study, the authors presented the bending of light reflection by employing an aperiodic graphene reflectarray [245]. The reflection phase of each unit cell is controlled by two graphene characteristics (i.e., chemical potential and the width of graphene strips). Using an appropriate graphene strip width and tuning the chemical potential through electrostatic bias results in phase discontinuity, eventually steering the reflected beam in the desired direction. In the end, all these concepts can be utilized in transmission through a transmitarray [217]. However, they are normally less appropriate for antenna applications because of the complicated fabrication process and the requirement of graphene multilayers to accomplish a broad phase with insignificant reflections [246,247]. The summary of the graphene leaky wave antennas, reflectarray antennas, and beam steering antennas are presented in Table 3.

### 5.2. Beam Steering Graphene Antennas and Phased Arrays

The functionality of common reflectarray antennas is limited to the static beam profile, and once the antenna is fabricated, the beam of the array can hardly be tuned. Thus, there is an increased demand for beam steering array antennas where the beam can be steered electrically through tuning the phases of each array unit cell [248]. Graphene provides beam tunability and beam steering ability in optical antennas because of its tunable electronic behavior with gate bias. Regardless of all their advantages, graphene-based antennas have less directivity and total efficiency because of their miniature size [249]. To tackle this issue, large arrays of graphene-based antennas could be designed to improve directivity and changing the phase of each array component with bias voltage could provide beam steering functionality. Chen et al. [250] demonstrated infrared beam steering using modulated surface plasmons over monolayer graphene. The beam steering is achieved by using a large number of gate pads beneath the graphene layer, which allows the switching of the gate voltage to tune the radiation pattern and its beam angle. The fabrication of such antenna is a complex task due to the large number of gate electrodes beneath the graphene sheet, but this large number of gate electrodes brings the advantage that the side radiation lobes can be sufficiently reduced. Nejad and coworkers investigated a tunable graphene high impedance antenna array at THz frequencies to realize the beam steering of the radiation pattern without using switches or phase shifters [251]. The authors designed a 2 × 2 graphene-based photoconductive array configuration and calculated the self and mutual impedance of the unit cell using HFSS software. The simulation results depicted that the array antenna had good input impedance at 0.8 THz, while when the chemical potential of the graphene was raised by 0.25 eV, the resonant frequency shifted to 1.2 THz. The beam steering of the radiation pattern of the investigated array antenna was modified by controlling the gate voltage at each graphene unit cell.

Recently the concept of the graphene-based phase shifter was employed in THz antenna arrays for steering functions [252]. A low-loss phase shifter controls the primary radiation beam of the THz array and provides a beam steering of 6°. The design of the phase shifter consists of two lines, characterized as a reference line and a delay line. The desirable amount of phase shift is achieved because of the difference between the path length of the two lines. A novel 3D beam reconfigurable antenna based on a graphene high-impedance surface is reported in [253]. The antenna has beam steering capability thanks to its loop geometry with a high-impedance surface. In addition, the switching of the radiation beam is attributed to the hybrid metal–graphene structure, where the beam steering property is controlled by tuning the graphene conductivity by applying the gate voltage. The increase in gate voltage makes the antenna capable of steering the beam in 25 directions by enabling its application in graphene THz communications. Furthermore, the beam-steering functionality is demonstrated for infrared imaging and sensing application using a graphene-dielectric metamaterial [254]. The author stressed the different design of the graphene-dielectric metamaterial by using a phase delay line, where the steering is manipulated by controlling the refractive index of the medium by changing the graphene conductivity. In the second design, they use an array of metallic plate waveguides of the same dimensions with the gap filled by graphene dielectric. The beam steering is achieved by tuning the effective refractive index of each parallel plate. Lastly, a design was made up of a combination of the two previous designs with the goal of utilizing the advantages of both symmetries by providing a wide range of output angles, and the absence of a metallic inclusion reduced the reflective losses and enabled the design to be completely reconfigurable.

## 6. Graphene Antenna Enhanced Photodetectors

Graphene is an alluring material that is mostly used in sensing and terahertz imaging devices for wideband photodetection [151,257]. As of late, it is commonly supposed that graphene could revolutionize the means for robust and cheap antenna integrated terahertz photodetectors functioning at room temperature based on Shur and Dyakonov approaches [48,258,259]. Graphene has the ability to sustain plasma waves which are feebly damped in excellent graphene samples. Moreover, they also possess extremely high transport mobility at room temperature [260]. In this way, multi-layer or single-layer graphene antenna integrated photodetectors quite surpass the conventional terahertz detectors. Over the most recent few years, different graphene-based antenna integrated geometries have been investigated for detection purposes, for example, single or multi-layer graphene–insulator–graphene heterostructures, graphene field-effect transistors, multiple cascaded graphene antenna assemblies, etc. [261]. Besides, some fascinating research studies validate the strong resonant detection by graphene insulator heterostructures in contrast to field-effect transistors [262,263]. The configuration of graphene top-gate antenna-coupled field effect-transistors has been employed to excite over-damped plasma waves for the broadband detection of terahertz frequency [264,265]. A few further examinations on analogous bow tie antennas have likewise accounted for the enhancement of coupling and responsivity in the terahertz band. Under other conditions, some fascinating outcomes were also obtained for the broadband terahertz detection ranging from 1.63 to 3.11 THz employing field-effect transistors, where the drain and source of the transistor act as an antenna for the approaching terahertz radiation. Besides, few photodetectors have been illustrated in the mid- and far-infrared regions [266,267,268,269].

Additionally, employing graphene in an antenna integrated photodetector enhances the responsivity and other characteristics of the photodetector, which have received intensive consideration. Moreover, its possible application has been deterred by compromises in the responsivity, functioning speed and bandwidth range of current graphene-based photodetectors. Semih and colleagues demonstrated the design of a photodetector comprised of gold–graphene patch nanostrips, which empower immediate wideband and highspeed photodetection by having high responsivity [270]. The nanostructures exploit graphene’s excellent properties (i.e., broadband optical absorption and high-speed photocarrier in graphene strips), thereby paving the way for the high-speed transport of photocarriers to gold resonators. Using this methodology, high responsivity is acknowledged without the utilization of tunneling barriers and quantum dots. The authors further stressed that by using a graphene gold antenna photodetector, a high responsivity is realized at 0.8 and 20 µm from infrared to visible regions with a photodetection speed greater than 50 GHz, thereby increasing the detection response time by nearly seven orders of magnitude compared to simple graphene-based quantum dots photodetectors. The concept of a graphene antenna sandwich photodetector is presented by Fang et al. for better photodetection properties. The geometry of the photodetector is composed of nanoscale circular antenna arrays, sandwiched between graphene layers [271]. An almost 800% enhancement in the photocurrent is observed by the conversion of infrared and visible photons into photons through graphene layers. The graphene sandwich antenna enhances the photocurrent in two different ways: by the transfer process of the generated hot electrons in the antenna geometry as the plasmons decay, and with the direct plasmon excitation of hot graphene electrons because of the antenna near field. Thus, the graphene antenna photodetector results in a 20% internal quantum efficiency at infrared and visible wavelengths. In another study, Yu Liu and coworkers focused on the absorption of graphene (i.e., 2.3% in visible and infrared regions), which limits graphene’s utilization in photodetectors [272]. Thereby, authors proposed that graphene along with metallic nanopillar antennas will result in higher light absorption in graphene-antenna-assisted photodetectors. Therefore, the free space EM waves are concentrated with the help of coupled antennas around the nanopillars by localized surface plasmon resonance, unequivocally affected by geomaterial structures. Furthermore, by tuning the geomaterial parameters of nanopillar antennas, tunable photodetector characteristics occur at a broad wavelength range of 0.6–1.2 µm with a high responsivity of 7 AW^−1^.

In a recent study, a novel concept for fast and efficient terahertz detection is demonstrated by using antenna integrated graphene p–n junctions [273]. Graphene is used as a photoactive material to reduce the current limitations of photodetectors (e.g., low speed and low sensitivity). The photo-thermoelectric effect is used to detect terahertz radiation. The authors further stressed that keeping a gap of 100 nm between the dipole antenna arms establishes a p–n junction, which serves to concentrate the incident terahertz radiation. The concentrated field thereby creates a photoresponse at the p–n junction. Applying voltage to the dipole arms of the antenna produces a graphene channel through the capacitive coupling, and thus the antenna acts as a photoactive area, providing enhanced optical absorption with strong field confinement. Furthermore, the proposed detector has a high sensitivity of 80 pW at room temperature, with a wide operating frequency bandwidth from 1.8 to 4.1 THz. Highly efficient photodetection is presented by Innocenti et al., where the authors proposed contacting a bow-tie antenna with graphene for high-speed room-temperature detection [274]. The effective photodetection is attained by engaging the array of bow-tie antennas with two different metal electrodes. The gap between bow-ties is contacted with graphene strips to improve the coupling and responsivity of incident light on the detector. However, the built-in confined electric field at the graphene–metal junction divides the excited electron–hole pairs. Thus, due to the contact with metal rods of different materials, the graphene strip (which is differently doped on either side) provides a responsivity of 34 µA/W at a frequency of 2 THz. In a related study, CVD-grown graphene field-effect transistors (GFETs) are integrated with a bow-tie antenna to make a terahertz detector [148]. The detector can rectify 0.6 THz signals at room temperature by using the field effect of a transistor, as when the incoming radiation is channeled to the drain and gate pads of the transistor, thereby the current produced by drain–source induces plasma waves. This effect can be described by the wave models of hydrodynamic plasma. A significant improvement of 14 V/W was achieved by exploiting GFETs along with a bowtie antenna in the terahertz range. In contrast to this, Capasso and colleagues studied metallic antennas in graphene photodetectors which served as a trapping assembly for incident light. By integrating the metallic antenna, the absorption cross section of light and photocarrier collection was improved. The overall responsivity of the graphene-antenna-assisted photodetector was improved by 200 times with 100% collection efficiency and a photoconductive peak gain of 2 dB [275].

**Figure 19:**
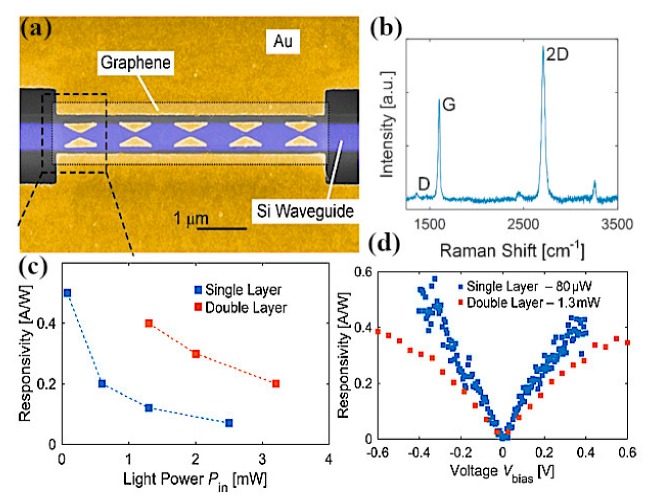
(**a**) Schematic of a waveguide graphene antenna integrated photodetector, where a long strip of graphene is placed beneath the bowtie antenna; (**b**) Raman spectroscopic measurement of a monolayer graphene photodetector; (**c**) Responsivity of the photodetector with respect to light intensity; (**d**) Photoresponse versus voltage of single- and bi-layer graphene detectors, where the double-layer detector has more responsivity than the single-layer photodetector. © 2018 ACS, Reprinted with permission from [277].

**Figure 20:**
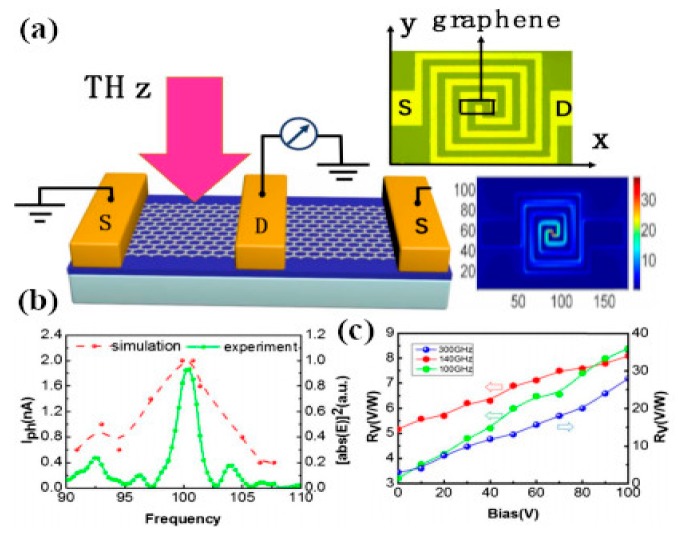
(**a**) Schematic of a graphene-antenna-assisted photodetector for terahertz detection at room temperature; the geometry of the proposed photodetector is comprised of three consecutive gold electrodes placed on the surface of a silicon wafer, with a graphene sheet placed between the two electrodes and contacted with them. Furthermore, a spiral antenna is placed beneath the graphene sheet which acts as coupler for incoming EM energy and provides an active channel for plasmons propagation. When the photodetector is illuminated through a terahertz laser the detector effectively couples EM energy, thereby providing fast detection. (**b**) Photoresponsivity simulation result comparison with experimental measurements; the experimental results agree well with the simulated results, having a maximum photocurrent response of 2 nA. (**c**) Responsivity of the detector at incident frequency with respect to electrical bias. © 2018 OSA, Reprinted with permission from [278].

The responsivity of graphene photodetectors is normally less due to the weak optical absorption of single-layer graphene. This problem is overcome by Pong Ma and coworkers through experimental investigations [276]. The authors suggest that using a waveguide integrated graphene photodetector could possibly enhance the plasmonic properties of graphene detectors (Figure 19a). They proposed a long strip of graphene with metallic nanoantennas for the enhancement of the electric field. However, surface plasmon polarization is excited in bowtie metallic nanoantennas by the transient electric field coupling to the graphene photodetector. The bowties are designed such that they concentrate SPPs in the gap between the bow-tie antenna arms with the confined electric field, while these SPPs interacting with the long graphene strip provide efficient absorption of terahertz light. Figure 19c,d depicts the responsivity with respect to single- and bi-layer graphene. Wan and coworkers presented and demonstrated the idea of integrating spiral antennas in graphene detectors for fast and efficient photodetection [277]. The schematic of the spiral antenna with the graphene photodetector is presented in Figure 20a. The detector geometry consists of three consecutive gold electrodes, which are contacted with a graphene sheet. The graphene sheet in between the electrodes along with the integrated spiral antenna serve as a coupler, by coupling EM energy to enhance efficiency and encourage photodetection through the hot-electron photo-thermoelectric effect. When the detector is illuminated by terahertz laser and biased through external voltage, a critical thermoelectric effect is observed with 28 V/W responsivity at room temperature. Tuning the frequency range of incident illumination gives a peak power density of 0.5 mW/cm^2^, and the maximum photoresponse is achieved at 100 GHz in the simulation, further validated through the experimental measurements depicted in Figure 20b. However, the responsivity gained by applying a voltage at the photodetector terminals (almost 28 V/W) is achieved at the resonance frequency, as shown in Figure 20c. The displayed outcomes suggest that it is a possible method for efficient terahertz detection, while the graphene antenna integrated photodetector provides great versatility and good performance that can be employed in optoelectronics for terahertz applications.

## 7. Graphene-Based Rectennas for Energy Harvesting Applications

The concept of solar energy harvesting through rectennas, converting solar energy to DC, has existed for nearly 40 years [278,279]. Rectennas in the microwave domain with conversion efficiencies greater than 70% have been exhibited. In contrast, rectennas operating at long infrared wavelengths use nanoantennas integrated with planar MIM diodes [280,281]. However, the present MIM diode designs are constrained in working frequency because of high RC response time and poor impedance mismatch with the coupled antenna, thus limiting the utilization for power conversion in the optical range. In the optical regime, rectennas are exploited to convert free space EM optical energy into direct current, having applications in low-power terahertz sensors and wireless communication devices [282]. Rectennas operating at optical wavelengths comprise nanoantennas coupled to fast diodes, as nanoantennas collect electromagnetic energy and coupled diodes convert the high-frequency AC field into DC power [283,284]. Because of technological constraints, harvesting practices for optical energy from terahertz to IR regimes are more difficult, due to the strong dispersive behavior of metal, the absence of high-frequency rectifying diodes with better conversion efficiency and impedance matching between the EM energy collector (antenna) and the rectifier diode. Impedance mismatching bounds the total output power collected by the antenna and transferred to rectifier diodes for DC conversion [285,286]. This issue is increasingly apparent in metal–insulator (MIM) diodes, having applications at terahertz or infrared wavelengths to realize optical rectennas [282]. Furthermore, the transfer of power between the rectenna elements is a serious issue restricting the conversion efficiency. However, it has been shown that in order to detect THz signals up to 3 THz, low-dielectric oxides offer better coupling efficiency between antenna and diode [287]. In contrast, the fundamental idea of an energy harvester is to give a noticeable value of rectified power, neglecting the bias voltage [288].

The presentation of graphene has escalated consideration for the enhancement of rectenna responsivity. Dragoman and Aldrigo recently worked on a graphene-based rectenna for efficient energy harvesting and obtained a higher conversion efficiency of 58.43% at terahertz frequency [289]. The authors proposed a solution integrating a metallic bowtie antenna with a Schottky diode for terahertz energy harvesting employing monolayer graphene on the top of an n-doped GaAs semi-conductor. The doped semiconductor and graphene layer provide a good matching condition for antenna and diode, thus improving the conversion efficiency. The harvesting of mid-infrared energy through different nanoantennas is presented by Ahmad et al. In their study they stress the conversion efficiency of different antenna designs, as spiral nanoantennas have a greater electric field than dipole and bowtie antennas [290]. Additionally, a bowtie antenna has the advantage of higher bandwidth, and through gap adjustment the electric field can be improved through the coupling of bowtie array elements. In another study, a realistic approach is demonstrated by employing a graphene nano-rectenna to harvest energy from human heat at around 30 THz infrared frequency bands [291]. almost A conversion efficiency of 53% is achieved by rectifying the collected electric field between the gap of bowtie antennas through graphene-based MIM diodes. Zhu and coworkers demonstrated an IR detector rectenna with high performance at room temperature employing graphene ultrafast diodes coupled to bowtie antennas [292]. The geometric diodes are dependent on the structural asymmetry along with the charge carrier path of graphene to deliver uneven current–voltage attributes. However, the planar geometry of graphene geometric diodes has the advantage of a femtosecond RC time constant, providing a basic necessity for the detection of high-frequency IR signals. The fabricated rectenna, employing graphene geometric diodes coupled to bowtie antennas, depicts strong optical behavior at 28 THz when the rectenna is excited by a CO_2_ laser at room temperature [293]. The maximum equivalent power of the rectenna at IR frequencies is 10^−9^ WHz^−1/2^ with the IR detection of 10^8^ cm Hz^1/2^W^−1^. Furthermore, the authors stress the utilization of the above-discussed rectenna IR detector in night-vision devices (e.g., vehicles), and the performance for the night-vision systems is calculated through the noise equivalent temperature difference (NETD). The NETD is the ratio of incident signal temperature needed to match with the detector noise, with the goal of the signal-to-noise ratio approaching one. For sensible car night vision it is necessary to have an IR identifier with an NETD value of 0.3 K [294]. In another study, the limitation of the limited power stored in the nano-batteries which supply power to the nano-sensors in a wireless body-centric network for healthcare systems is overcome by using graphene rectennas to provide constant power for the operation of the nano-sensors [295]. The rectenna model proposed by Lu and coworkers is composed of a graphene bowtie antenna coupled to graphene geometric diodes capable of harvesting IR frequency and producing a DC current in the absence of any external source. Furthermore, the graphene nano-rectenna is compared with a state-of-the-art carbon nanotube (CNT) rectenna and a piezoelectric nano-generator, and the graphene rectenna outperformed the CNT rectenna by producing a higher voltage, thus proving itself as an efficient power source for nano-sensors in body-centric applications.

**Figure 21:**
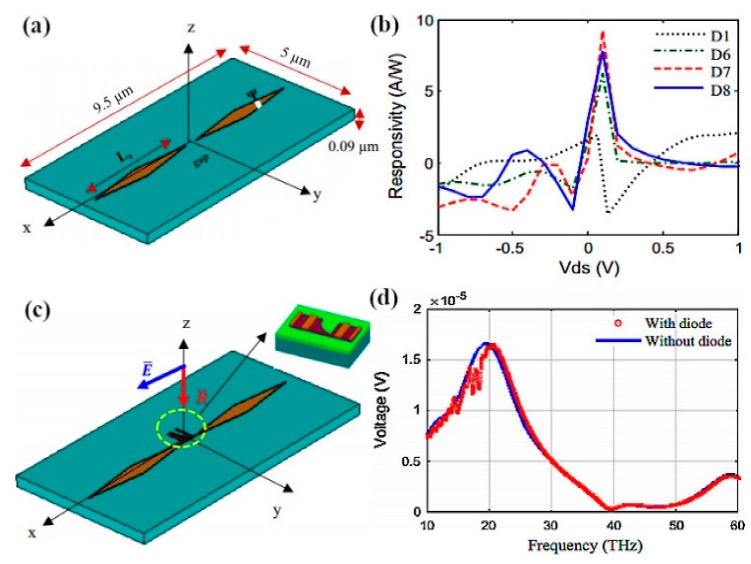
Graphene rectenna. (**a**) Schematic of a terahertz antenna designed to operate at terahertz frequency. (**b**) Responsivity of the graphene geometric diode with varying geometric parameters. (**c**) Schematic of a graphene rectenna coupled to a graphene geometric diode and placed between the gap of the antenna. (**d**) Voltage of the rectenna when the diode is inserted between the antenna gap; the rectenna is able to rectify a wide infrared frequency range. © 2018 Springer, Reprinted with permission from [299].

Additionally, the enhancement of the energy harvesting of a coupled nanoantenna with a geometric diode is demonstrated by employing a transmitarray to harvest infrared energy at 19 THz. Two key methods are proposed for the improvement of the obtained voltage through a graphene nanoantenna by Araby et al. [296]. The first method is to focus electromagnetic waves by means of a transmitarray on the surface of a graphene nanoantenna coupled to geometric diodes; by employing this method, an increase of the received voltage from 16.5 to 97.6 µV is realized. However, in the second method, Yagi metal antennas are utilized to improve the directivity of array elements coupled to a transmitarray [297]. In a subsequent study by the same authors, different geometries are employed for graphene geometric diodes in order to enhance the responsivity of the IR rectenna [298]. A Monte Carlo simulation technique is adopted to analyze the I–V characteristics of the graphene geometric diode. The prosed rectenna depicted in Figure 21a can harvest infrared energy at 20 THz. The structure of the graphene diode is designed using a graphene sheet placed on top of a SiO_2_/Si substrate, and metal contacts for external terminals are placed on top of the graphene. A change in responsivity occurs by varying the geometric parameters of the graphene diode, as shown in Figure 21b. However, a peak voltage of 1.5 V is attained at a wide terahertz frequency bandwidth when the diode is coupled to an antenna, as illustrated in Figure 21c. Additinlly, the summary of key graphene rectennas are given in Table 4.

## 8. Graphene-Based Antennas for Plasmonic and Biosensing

The localized surface plasmon resonance of optical antennas enables different sensing applications (i.e., biosensing, plasmonic sensing (LSPR) and label-free sensing) due to the high enhancement of electric field and sharp resonant excitation of the metallic structures. However, in visible and infrared regimes, these metals have higher losses due to the lack of interband absorption. Advancements have been made to achieve excellent sensing properties by using graphene–metal hybrid resonators for such applications. Graphene-enabled silver nanoantenna sensors are designed to improve the sensitivity of sliver nanoantenna sensors [299]. An overall 2600% higher sensitivity is acquired when a silver nanoantenna is integrated with graphene. Additionally, the graphene layers provide protection for silver nanoantenna structures to protect them from sulfidation, as silver nanoantennas without graphene can react with atmospheric sulfur, forming a tarnish and roughening the surface of Ag_2_S, which decreases the sensitivity. A Fano metamaterial with dynamic reconfiguration abilities is employed for switching and sensing [300]. The reconfigurability of the metamaterial is enabled through electrically tuning graphene, and the metamaterial has a special type of effect known as electromagnetically induced transparency when destructive interference is taking place between the narrow- and broad-band dipolar plasmons. The ultrahigh sensing of infrared radiation is investigated through a graphene-based broadband THz detector at room temperature with a very short sensing time of 50 ps [301]. Meanwhile, terahertz graphene sensors based on double Fano resonance achieved a sensitivity of 6.52 and the sensing range is controlled by the Fermi energy of graphene [302]. Nejad et al. proposed a very interesting concept for label-free sensing using a hybrid graphene–molybdenum disulfide-based ring resonator [303]. The sensitivity enhancement is acquired by placing a hybrid graphene, which also leads to the attainment of a high figure of merit with high sensor performance. In a subsequent work by the same researchers [304], a staking symmetry of a silicon nitride dielectric resonator ring is vertically coupled to graphene strip resonators for enhancing the interaction of surface plasmons and the target molecules.

The mid-infrared region is especially appropriate for biosensing, as it includes the atomic vibrations that interestingly recognize the biochemical nature of molecules such as proteins, lipids and DNA. Among various techniques, IR absorption spectroscopy is a ground-breaking method that gives precise biochemical information in a non-destructive label-free style by getting vibrational patterns. Nevertheless, the vibrational absorption signals obtained from IR spectroscopy are restrictively weak on account of an enormous mismatch between the mid-IR wavelengths and biomolecular sizes. To reduce these hurdles, high sensitivity can be accomplished by manipulating strong optical near-fields in the vicinity of the graphene–metallic resonant structures. In particular, biosensing is a research area where graphene tunability and strong IR light confinement offer excellent prospects. Additionally, the spatial control of graphene plasmons in the IR region makes them very appealing for enhanced light–matter interactions and IR nanophotonics.

**Figure 22:**
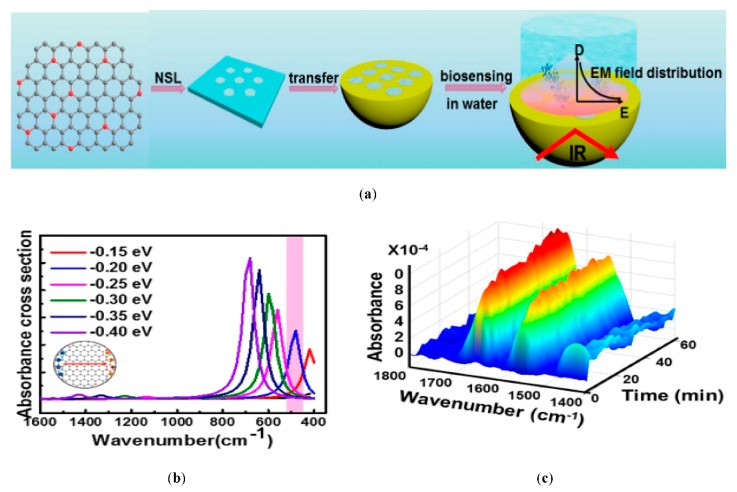
(**a)** Schematic illustration of a graphene-based biosensor in the IR range; (**b**) Resonance tuning of the sensor with graphene Fermi energy tuning; (**c**) SEIRA spectra as a function of the immunoreaction of the boron-doped graphene nanodisk array. © 2018 American Chemical Society. Reprinted with permission from [307].

Rodrigo et al. demonstrated mid-infrared plasmonic biosensing with an integrated graphene resonator using infrared spectroscopy [305]. The weak interaction of infrared light with nanometric biomolecules is enhanced by exploiting graphene’s unique electro-optical properties. The authors experimentally demonstrated the high sensitivity of the plasmonic tunable biosensor by tuning the graphene’s conductivity with varying voltage in the range from 0 to 120 V. Moreover, the extreme confinement of graphene plasmons enables superior sensitivity in detecting the refractive index and vibrational fingerprints of nanometric molecules and achieved two orders of magnitude greater localization of infrared light with respect to metallic antennas. Thereby, the two advantages (enhanced sensitivity and spectral reflectivity) of the plasmonic biosensor with a graphene resonator open exciting prospects for biosensing and graphene biosensors. Most recently, Zheng and coworkers worked on a graphene-plasmons-enhanced IR biosensor for the detection of aqueous-phase molecules (Figure 22a), where the graphene-based plasmonic sensor is placed on a layer of biomolecules and the detection is carried out through attenuated total reflection mode [306]. The sensor is fabricated using a boron-doped graphene nanodisk array which supports totally surface enhanced infrared absorption (SEIRA) spectroscopy. The plasmonic resonance is also tuned by different Fermi energies to improve the tunability of the sensor, as presented in Figure 22b. The specific protein recognition is evaluated by utilizing the near-field enhancement of graphene plasmons, acquiring a maximum detection of 0.5 nM for the target protein obtained from the surface enhanced infrared absorption (SEIRA) spectra as shown in Figure 22c.

The enhanced mid-infrared sensing using the concept of total optical absorption is investigated in [307] by analyzing a graphene-based metamaterial biosensor to acquire tunable plasmon-induced transparency (PIT) in IR wavelengths as summarized in Table 4. The PIT effect can be easily modified by tuning the graphene metamaterial conductivity with varying chemical potential. Additionally, the tunable performance of the metamaterial sensor provides strong optical sensing with the PIT effect. The authors further extended their investigation to biosensing applications, where they proved that biosensing is highly dependent on the ultra-thin buffer layer between graphene and the substrate layer. Moreover, graphene-based nano-aperture antennas can provide enhanced bio sensitivity and accuracy with cross-shaped slots in the antenna sensor geometry [280]. The reconfigurability in subsequent work by the same authors is realized by employing fractal geometries [281] in the graphene-based nano-aperture antennas which have the advantage of confined electric fields, thus achieving greater sensitivity than the previous one. The graphene-based antenna sensors are not only limited to biosensing, but various studies have also reported flexible graphene-based antenna sensors for wearable technologies [308]. The high mechanical strength of graphene provides enhanced performance stability under bent conditions for medical and gas sensing applications. The idea of a graphene nanoflakes printed antenna for low-cost RFID sensing applications is demonstrated in [309], where the sensor is constructed from a dipole graphene antenna on a flexible substrate which provides flexibility during operation with the total efficiency of 32%. In another work, RFID sensors based on a graphene oxide antenna is employed for wireless humidity sensing by providing an efficient way to detect humidity changes without using a battery source. This type of sensor is in great demand for Internet of things applications where low-cost efficient sensors are normally required for monitoring.

## 9. Synthesis of Graphene and the Fabrication of Graphene–Metal Plasmonic Nanoantennas

Enormous efforts have been made in recent decades towards the synthesis and fabrication of graphene devices and graphene integrated optoelectronics. There are generally three main methods to synthesize graphene layers, which are arranged into diverse groups because of their different synthesis procedures—that is, mechanical cleavage (MC), epitaxial growth (EG) and chemical vapor deposition (CVD) are the well-known methods for the fabrication of graphene. Graphene is a single layer of carbon atoms and can be extracted from bulk graphite through mechanical cleavage with the help of adhesive tape. In 2005, Novoselov et al. synthesized the first in-lab graphene monoatomic layer through the MC method and experimentally achieved maximum carrier mobilities of up to 10^6^ cm^2^.V^−1^.s^−1^ [310,311]. Although MC is the most advantageous method for the production of high- grade graphene, the long time required for the exfoliation procedure and the monoatomic size restrict the huge-scale production of graphene through MC. Subsequently, the artificial growing of monolayer graphene has attained extraordinary significance throughout the world. The epitaxial growth of graphene utilizes silicon carbide as a source material. At high temperature (1000 °C), silicon normally starts thermal evaporation leaving behind graphene sheets on the catalytic surface [312,313]. The estimated mobility of graphene produced through EG at room temperature ranges from 1 × 10^4^ cm^2^.V^−1^.s^−1^ to 3 × 10^4^ cm^2^.V^−1^.s^−1^ [314,315,316,317,318]. Nevertheless, silicon carbide is generally utilized as a ground substrate in high-speed optoelectronics. The immediate development of monolayer graphene on silicon carbide bypasses the need of extra substrate growth and this is beneficial for graphene use in silicon carbide electronic components such as LEDs and ultrahigh-frequency transistors [319,320]. However, special consideration must be kept throughout the EG growth process because the non-self-limiting nature of thermal decomposition and the high cost of silicon carbide wafers restrict the huge production of pure monolayer graphene on the surface of silicon carbide [321]. To accomplish economical and huge-scale graphene production, chemical vapor deposition is the most suitable and effective graphene fabrication method. CVD fundamentally practices the decomposition of carbon atoms from a hydrocarbon on some metallic catalyst surface and results in structures such as uniform hexagonal lattices of carbon atoms, thus producing monoatomic thick graphene layer atoms [322,323,324]. In general, methane is used as the hydrocarbon along with copper as the metallic surface. However, in 2009, CVD was utilized for the first time to grow large-area monolayer graphene and experimentally observed fluctuating carrier mobilities ranging from 1.64 × 10^4^ cm^2^.V^−1^.s^−1^ to 2.5 × 10^4^ cm^2^.V^−1^.s^−1^ were achieved after transferring the monolayer graphene from the metallic copper surface to the insulated wafer [325]. Additionally, the CVD process is practically self-sufficient, and thus the CVD process will routinely stop when single-layer graphene is formed on the whole catalytic metal surface. Consequently, large graphene sheets with a uniform layer can be obtained through this process. In contrast to the above discussion, the thermal expansion coefficient difference between the graphene and copper can introduce defect wrinkles on the graphene sheet, which significantly reduces the quality of the graphene by having reduced mobilities in contrast to other fabrication methods [325,326]. In the interest of producing high-grade and economical graphene, additional development should be accomplished in CVD processes (e.g., plasma CVD and atmospheric low-pressure CVD) [326,327,328,329]. These new techniques also reduce the growth time and can effectively control the growth of stacked graphene layers [330].

**Figure 23:**
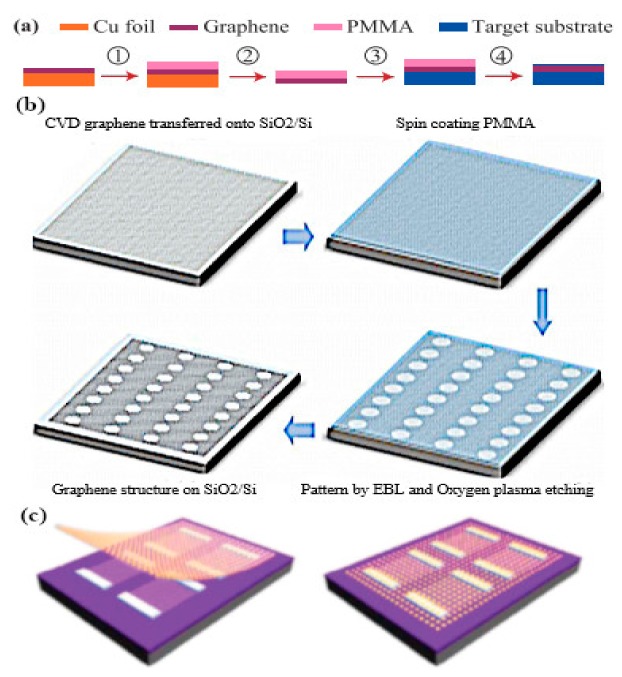
(**a**) Graphene transfer procedure schematic. (**b**) Patterning and fabrication of graphene antenna structure; the substrate with graphene is spin-coated with a PMMA layer then patterning is done through EBL and excess graphene is etched away through oxygen plasma. (**c**) Fabrication of graphene hybrid metal antenna; the metal is deposited when patterning is done, thereby integrating graphene with metal, providing a graphene–metal antenna. © 2017 PIER, © 2018 Springer, © 2011 Macmillan Publishers Limited. Reprinted with permission from [311,318,325].

The fabrication of graphene–metal hybrid antennas and antenna integrated optoelectronic devices require various steps, that is, graphene synthesis, transfer to the proposed substrate, patterning of nanostructures through lithography techniques and lastly metal etching and liftoff. Firstly, a graphene layer is grown using CVD on catalytic metal, with the aid of poly(methyl methacrylate) (PMMA). The most critical part is the transfer of graphene to the target substrate and ensuring that the graphene is not damaged, as depicted in Figure 23a. The three most common techniques utilized for transfer process are carrier film, stamp and self-release [331]. The carrier film is discussed here as taking part in four different stages. Initially, a metal-containing graphene layer is subjected to spin coating by putting some drops of PMMA on the graphene surface; this is followed by etching the metal surface using a specific etchant. Afterward, the PMMA/graphene film is applied to the proposed substrate (mostly to silicon wafers in the case of plasmonic antennas); the schematics of the transfer process is shown in Figure 23a. In the last step graphene is left on the substrate by dissolving the adhesive layer of PMMA through organic solvent. However, to complete the transfer process, a water wash is necessary to remove synthetic buildups formed during the transfer process, and the substrate containing graphene must be heated for several hours to completely remove the water particles and provide good adhesion between the graphene and substrate. In contrast, for graphene-only nanoantennas, the spin-coated PMMA mask is exposed to electron beam lithography (EBL) to pattern the graphene structures, followed by exposure to oxygen plasma to remove unnecessary graphene, thus simultaneously forming graphene antenna structures [310] (Figure 23b). However, after the transfer process, the fabrication of metallic structures on top of the graphene sheet begin. This can be done by utilizing different patterning procedures (i.e., electron beam lithography or ion beam lithography). The first lithography technique is applied to a photoresist layer, which has been spin-coated on the substrate for patterning nanostructures [332]. Next, the metal is deposited on the substrate containing graphene through electron beam evaporation and the excess metal is etched through the lift-off process as shown in Figure 23c. Furthermore, if the antenna is sandwiched between the graphene sheets then the hybrid graphene–metal antenna would be subjected to a CVD process again to grow a graphene layer on top of the metal [333,334]. The advancement of CVD empowers the creation of large-area graphene at microwave frequencies with dimensions equal to microwave wavelengths, thus enabling the utilization of graphene in the microwave region. Prior to this, the vast majority of works were aimed at the simulation of graphene antennas and frequency-selective surfaces [209,335,336]. In any case, as mentioned previously, the graphene produced through CVD cannot reach the desired specifications at the moment, thus making it difficult for the realization of theoretical modeling and simulation that utilize a conductive surface of graphene.

Although the huge advances in the patterning of leading materials have provoked the improvement of microstructures [337], the lithography and CVD techniques are costly, time-consuming and highly complicated for the fabrication of large-area graphene structures [338,339]. Hence, the development of low-cost and convenient methods for graphene patterning has attracted more consideration. Inkjet printing has been broadly researched as a possible strategy for patterning graphene structures on substrate materials. It is worth mentioning that inkjet printing utilizes conducting inks and the conducting ink should meet some specific requirements to be printed through a nozzle (e.g., viscosity and surface tension). Jeng and coworkers initially provide experimental evidence for printing a graphene sheet utilizing the reduction of graphene oxide ink [340]. Moreover, inkjet printing is most effective for the fabrication of multilayer graphene by reducing graphene oxide without the addition of any additives. Experimental results of an inkjet-printed dipole antenna validate the printing of graphene by maintaining good electrical conductivity and the sheet resistance can be actively controlled by manipulating the printing pattern. In a recent study an RFID antenna for wearable applications is fabricated through 3D printing using graphene ink [341]. Water-based graphene ink (HDplas^®^ IGSC02002) is used for printing a multilayer graphene patch antenna through direct-write dispensing. In the end, the graphene antenna is baked at 60 °C for half an hour, which provides better adhesion with the substrate material [342]. The fabricated antenna has a stable radiation pattern and good beam reconfigurability at microwave frequencies. Thus, graphene printing offers an excellent approach for the fabrication of antenna devices operating in the microwave region. Subsequently, graphene has indicated incredible potential in functional devices and guaranteed practical applications.

## 10. Conclusions

Graphene signifies an emergent outpost that unites the various research fields of physics, terahertz plasmonics, optoelectronics, nanophotonics and material science. The remarkable properties of graphene have exposed stimulating prospects in these photonic fields, where novel innovation and finding new solutions to current problems are still evolving. From the perspective of the electronic devices and photonics systems, graphene is a 2D monoatomic layer of carbon atoms with an inherently passivated surface. As the production of this material drives the present market for graphene utilizations, there is a clear hierarchy in how soon the graphene applications will influence the consumer. In the near future, the manufacturing methods for this material will evolve and provide the possibility of accessing graphene materials with low costs for applications utilizing a low grade of graphene. Most likely, in future years, the devices requiring the highest grade of pure graphene of electronic quality will take time to develop and improve the graphene synthesis method. The high mechanical properties of graphene will bring a drastic change in current flexible device technologies, such as solar cells and various other plasmonic devices for imaging and sensing applications.

To make an impact on industry-standard device utilizations, the current demanding challenges of the graphene must be overcome. Amongst many others, the huge-scale synthesis of high-quality graphene is of foremost significance, which will have a huge impact on this field. Precisely, developments in graphene can considerably extend the life of graphene plasmons, which is highly associated with the device operation characteristics like absorbance, modulation efficiency, strong light interaction, sensitivity and more. Additionally, quantum and nonlinear effects present in profound subwavelength graphene nanostructures will be extensively explored in the future, which will provide a new stage for quantum information and communication. The unique optical properties of graphene-based optical antennas and graphene antenna integrated optoelectronics will shift the indicative paradigm of nano-optoelectronics. On a more extensive scale, the impact of the incorporated graphene optical antennas on numerous innovations, including ultra-fast photodetectors, nano biosensors, and ultra-fast optical chip interconnects is forthcoming.

In this article, a review is presented on the development of graphene-based plasmonic antennas, from their origin to the current situation. Their deficiencies and operating abilities have discussed in detail. Three basic themes stand out in this survey: frequency tunability, graphene integration with noble metal antennas and miniaturization. These are the most remarkable and alluring highlights of graphene, and they are the foundation that enabled the research community to realize graphene tunable antennas, graphene antenna photodetectors and modulators, graphene antenna biosensors, and energy harvesters. Even though the realization of these devices is so difficult, the recent improvements in the fabrication process of graphene promises a bright future. From the theoretical point of view, we believe that research in graphene nanoantennas will pursue a few directions in the coming years, such as the merging of graphene antennas with metamaterials. These directions will lead to a proliferation in both theoretical investigations and practical industrial products for high-speed terahertz communication, terahertz imaging, bio-sensing, photodetectors and energy harvesters.

## Figures and Tables

**Table 1:** Summary of the various design performances and their contributions discussed in Section 3.

Contribution/Study	Design and Resonant Frequency (THz)	Substrate Material	Bandwidth (THz)	Tunability Range (THz)
Graphene-based nanoantenna-enhanced photomixer for wideband-tunable terahertz frequency generation, 2013 [122]	Graphene nanoemitter for planar emission @ 392	SiO_2_/Si	25	380–400
Lateral confinement of SPP in graphene nanoribbons antenna, 2013 [106]	GNR on flat metallic surface @ 120	SiO_2_	50	100–600
Graphene cylindrical waveguides as reconfigurable antennas, 2015 [113]	GCW-based dipole antenna @ 1.5	SiO_2_	0.3	1–3
Waveguide fed tunable graphene–metal antenna for sensing, 2016 [138]	Staked graphene patch with waveguide fed @ 2.73	Si/SiO_2_ substrate with Ag	0.8	2.63–2.73
Efficiency improvement of graphene stack antenna with LIM and high index material layers, 2017 [102]	Graphene-based stack dipole antenna @ 1.2	LIM and HIM Stacks	0.6	0.5–3
Reduction of mutual coupling of graphene antenna arrays, 2017 [105]	Graphene antenna array @ 1	SiO_2_	8	1–8
Tuning of radiation characteristics, 2017 [112]	Graphene patch antenna @ 0.97	SiO_2_	1	0.97–1.09
Electrically tunable polarizer with graphene cross antenna, 2018 [133]	Graphene loaded cross shape dipole antenna array @ 50	SiO_2_	67	33–100

**Table 2:** Summary of graphene–metal antennas and their contributions as discussed in Section 4. LSPR: localized surface plasmon resonance.

Contribution/Study	Geometry and Wavelength	Results and Measurement
Control of graphene plasmons, 2014 [185]	Metallic dipole wrapped in graphene sheet @ 11.06 µm	Enhancement factor *f^2^* = 3
Tuning of scattering response of nanoantenna, 2014 [140]	Graphene dipole @ 500 nm	Multi-layer graphene results in better scattering
Magnetic tunable responses of graphene, 2015 [172]	Graphene diabolo with Au reflector @ 7 µm	Intensity modulation 1460%, tuning range 63.1%
Active modulation of visible light with graphene, 2016 [169]	Graphene with silver rod array @ 749 nm	Reflection (>600%) and modulation depth 36%
Beam steering and far-field light propagation modulation, 2017 [144]	Graphene metasurface with Au resonators @ 8.4 µm	98% absorption at *E_f_* = 0.25 eV with better beam intensity
Tunable metasurface for metallic biosensor antenna, 2017 [184]	Rectangular Au arrays with graphene @ 7142 nm	Enhancement factor from 13 to 20, at a rate of ~0.07/cm^−1^
Broadband modulation of terahertz waves, 2017 [175]	Bowie arrays on top of VO_2_ film @ 199 µm	Maximum intensity 1407 J/m^3^
LSPR of nanorods with graphene for bio-sensing, 2018 [139]	Meta-graphene double-nanorods @ 10 µm	Extinction cross section 20 µm^2^, directivity 24 dB

**Table 3:** Summary of graphene leaky-wave and reflectarray antennas as discussed in Section 5. FPCA: Fabry–Perot cavity antenna; OAM: orbital angular momentum.

Contribution/Study	Geometry and Frequency	Phase Tuning and Beam Scanning
Tuning beam steering with HIS, 2015 [179]	Leaky-wave graphene antenna @ 3.2 THz	60° scanning angle, reflection phase 135°
Dispersion and radiation properties, 2016 [211]	Substrate–superstrate graphene LWA @ 0.92 THz	Beamwidth 0° to 45°, Chemical potential tuning 0 eV to 1 eV
Efficiency improvement of reconfigurable LWA, 2017 [214]	Graphene FPCAs with metasurface @ 1 THz	30° and 45° beam scanning, 70% efficiency
Dynamic reconfiguration of vertex modes, 2016 [255]	Graphene reflectarray with circular sectors @ 1.6 THz	Beamwidth 24.6°, efficiency 67%, reflection phase 360°
Sinusoidal modulation for beam scanning, 2017 [205]	Leaky-wave antenna with gapped graphene @ 2 THz	45° beam scanning, 77% radiation efficiency
Generation of OAM vortex waves, 2017 [256]	Graphene metamaterial reflectarray @ 1.8–2.8 THz	360° phase range, reflection magnitude -2.5 dB
Pancharatnam–Berry phase principle, 2018 [242]	Reflectarray using graphene @ 1.52 THz	Tunable phase range of 360°, beamwidth 120°

**Table 4:** Summary of the literature discussed in Section 6, Section 7 and Section 8.

Contribution/Study	Geometry and Method	Measurements
Low-cost graphene antenna fabrication, 2015 [342]	Microstrip graphene antenna @ 890 MHz, ink-printed graphene	Conductivity 1.25 × 10^4^, sheet resistance 1.9 ± 0.1
Fabrication of graphene antenna, 2016 [334]	Graphene hexagon antenna fabrication on SiO_2_ @ 0.1–10 THz	CVD and focused ion beam lithography method
I–V characteristics of a graphene rectenna, 2017 [297]	Graphene geometric diode coupled with nanoantenna @ 20.5 THz	Voltage 1.6 × 10^−5^ V, responsivity 1.646 A/W
Energy harvesting enhancement, 2017 [296]	Nanoantenna coupled to graphene diode @ 19.4 THz	Received voltage 97.6 µm, gain 20 dBi
Ultra-sensitive near-infrared photodetection, 2017 [273]	Metallic nanopillar antennas with graphene @ 0.6–1.2 µm	Photoresponsivity 7 A/m at 8 µm, absorption 70 % at 8 µm
Highly-responsive ultrafast photodetection, 2018 [270]	Gold-patch graphene nano-strips @ 0.8 µm, 20 µm	Responsivity 0.6 A/M, 11.5 A/M, output power -80 dBm
Nano-rectenna for body-centric networks, 2018 [295]	Insulator-coated carbon nanotube cathode with bowtie nanoantenna	Capacitor charge time 2.2 s, capacitance 100 mF-cm^-2^
Graphene geometric diode IR harvesting, 2018 [297]	Nanoantenna coupled with geometric diode @ 20.5 THz	Current 15 mA, responsivity 9 A/W
graphene-plasmons IR biosensor, 2018 [307]	boron-doped graphene nanodisk array @ 400-800 (cm^-1^)	Absorbance 8× 10-^4^ at -0.4 eV, maximum detection 0.5 nM
Meta biosensor, 2018 [308]	Slotted metametrial @ 16-22 THz	Transmittance 0.2 at 18 THz
CVD graphene synthesis, 2018 [320]	CVD precursors H_2_, CH_4_, and pure copper, pressure 760 °C	Raman shift 2680 cm^−1^
Fabrication of large-area graphene, 2018 [332]	Circular and rectangular graphene nanomesh, EBL method	Photoconductive effect 10 mA/W, bandgap 0.23 eV
Fabrication of graphene-assisted biosensor nano slot antennas, 2019 [312]	Monolayer graphene growth through CVD and focused ion beam lithography	D peak 1350 cm^−1^, 630 E-field enhancement
Plasmonically enhanced photodetector, 2019 [274]	Graphene waveguide with bowtie antenna @ 1480–1620 nm	Responsivity 0.5 A/m, photoresponse speed 110 GHz
Fast and sensitive terahertz detection, 2019 [273]	Antenna integrated graphene p–n junction @ 1.8–4.25 THz	Speed 30 ns, noise equivalent power 80 pW/$\sqrt{\mathrm{Hz}}$
